# Caging tin oxide in three-dimensional graphene networks for superior volumetric lithium storage

**DOI:** 10.1038/s41467-017-02808-2

**Published:** 2018-01-26

**Authors:** Junwei Han, Debin Kong, Wei Lv, Dai-Ming Tang, Daliang Han, Chao Zhang, Donghai Liu, Zhichang Xiao, Xinghao Zhang, Jing Xiao, Xinzi He, Feng-Chun Hsia, Chen Zhang, Ying Tao, Dmitri Golberg, Feiyu Kang, Linjie Zhi, Quan-Hong Yang

**Affiliations:** 1 Nanoyang Group, School of Chemical Engineering and Technology, Collaborative Innovation Center of Chemical Science and Engineering (Tianjin), Tianjin University, Tianjin, 300072 China; 2 CAS Key Laboratory of Nanosystem and Hierarchical Fabrication, CAS Center for Excellence in Nanoscience, National Center for Nanoscience and Technology, Beijing, 100190 China; 3 Engineering Laboratory for Functionalized Carbon Materials, Shenzhen Key Laboratory for Graphene-based Materials, Graduate School at Shenzhen, Tsinghua University, Shenzhen, 518055 China; 4 International Center for Materials Nanoarchitectonics (WPI-MANA), National Institute for Materials Science (NIMS), Namiki 1-1, Tsukuba, Ibaraki, 3050044 Japan; 5 Queensland University of Technology (QUT), 2 George St., Brisbane, QLD 4000 Australia; 6 School of Marine Science and Technology, Tianjin University, Tianjin, 300072 China

## Abstract

Tin and its compounds hold promise for the development of high-capacity anode materials that could replace graphitic carbon used in current lithium-ion batteries. However, the introduced porosity in current electrode designs to buffer the volume changes of active materials during cycling does not afford high volumetric performance. Here, we show a strategy leveraging a sulfur sacrificial agent for controlled utility of void space in a tin oxide/graphene composite anode. In a typical synthesis using the capillary drying of graphene hydrogels, sulfur is employed with hard tin oxide nanoparticles inside the contraction hydrogels. The resultant graphene-caged tin oxide delivers an ultrahigh volumetric capacity of 2123 mAh cm^–3^ together with good cycling stability. Our results suggest not only a conversion-type composite anode that allows for good electrochemical characteristics, but also a general synthetic means to engineering the packing density of graphene nanosheets for high energy storage capabilities in small volumes.

## Abstract (web summary)

The excessive porous space in carbon anodes for lithium-ion batteries has to be utilized for high volumetric performance. Here the authors show an adaptable sulfur template strategy to yield graphene-caged noncarbon materials with a precisely controlled amount of void, enabling ultrahigh volumetric lithium storage.

## Introduction

Because of their high energy density and environmental friendliness, lithium-ion batteries have become one of the most important energy storage devices with wide applications in portable electronic devices, electric vehicles and grid energy storage systems. Considering the continuing demand for the miniaturization of electrochemical energy storage devices, which means storing as much energy as possible in limited space, volumetric energy density has become a critical parameter, but rarely emphasized in earlier studies of lithium-ion batteries^1,2^. Currently, the conventional graphite anodes are limited by their relatively low theoretical capacity^3^. Meanwhile, research on the promising next-generation noncarbon anode materials, such as tin (Sn) and silicon (Si)-based materials, has been mostly focused on improvements in the gravimetric capacity and the cycling performance. In fact, noncarbon anode materials also possess a huge advantage in volumetric performance over carbonaceous anodes due to their much higher gravimetric capacity and compact density^4–9^. Regrettably, the volumetric capacity has not received much attention and, even worse, their volume expansion in lithiation severely restricts real volumetric performance^10–13^.

**Fig. 1:**
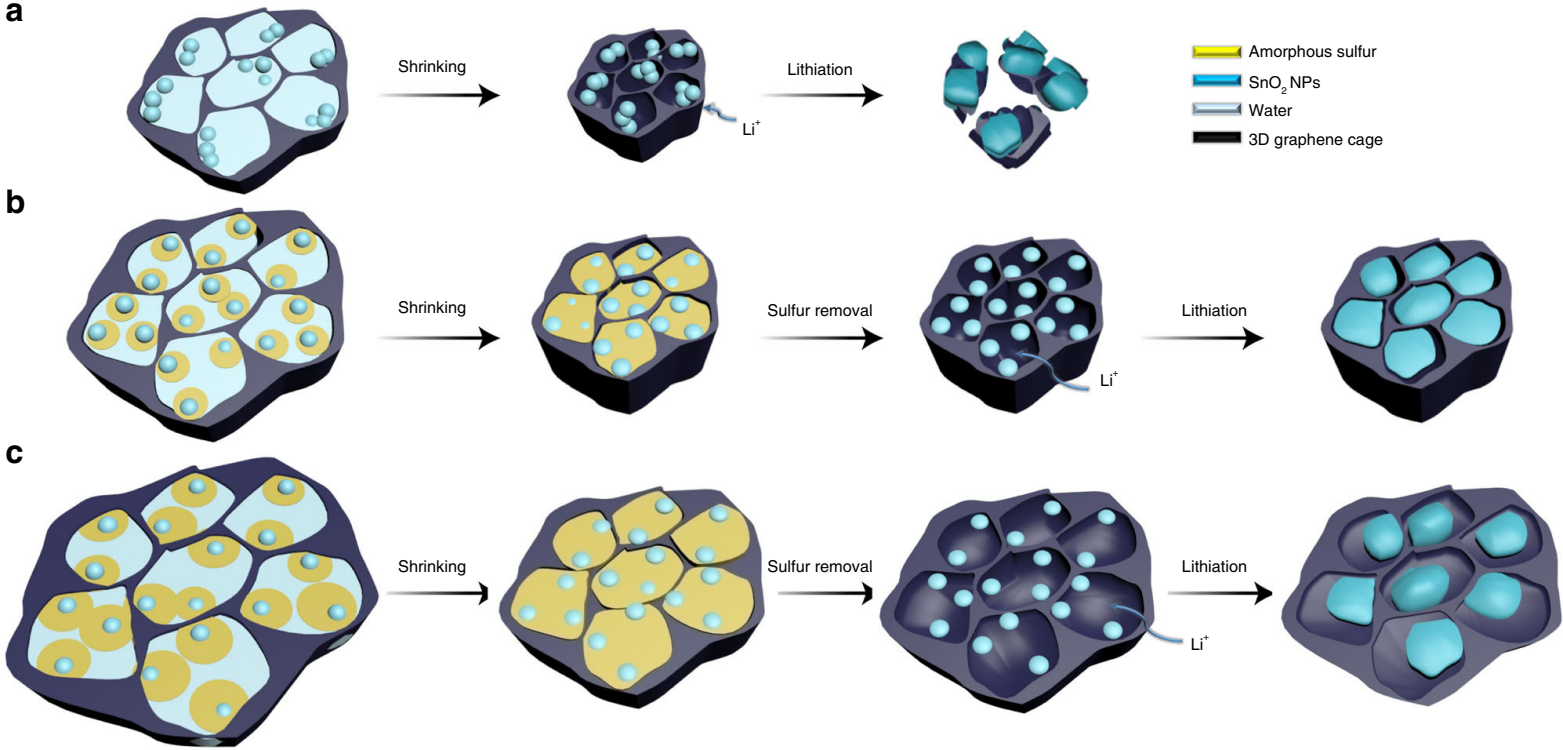
Sulfur template control of incorporated void space. **a** Schematic of the limited space produced after the shrinkage of a 3D graphene assembly, leading to cracking of the graphene cage and electrode pulverization when the SnO_2_ expands during the lithium storage process. **b** When an appropriate amount of sulfur is used as a sacrificial template for incorporating void space, the SnO_2_ has enough space for lithiation. Note that sulfur removal could result in the production of the smallest sufficient void space, thus achieving high volumetric performance. **c** The removal of excess sulfur would introduce a large void space and compromise the volumetric capacity

Carbon materials play an important role in lithium-ion batteries not only directly as electrode materials^14,15^, but also as conductive networks^16^ and electrochemical reaction frameworks for the loading of active materials^17^. To address the problem of volume expansion, carbon-noncarbon hybrid structures (hierarchical^18,19^, core-shell^20^, sandwich-like^21^, array nanostructures^5,22,23^ and carbon cages^12^) have been extensively studied. The carbon in them is used to construct voids that buffer the volume expansion of the noncarbons and provide a shell for stable solid-electrolyte interphase (SEI) formation^24^. In addition, the carbon increases the electrical conductivity which improves the utilization of the noncarbon materials^17^. However, in most cases such as illustrated in Fig. 1c, these structures introduce excess void space which counters the attempt to obtain a high volumetric-specific capacity. Some progress has been made in the design of voids to address this problem. In this respect, in situ synthesis offers a greater advantage over the traditional ex situ synthesis route^25–27^, which suffers from the low-efficiency use of void space with noncarbon components filling a stiff carbon network. However, unsuitable voids for the noncarbon component always exist due to the roughly designed and controlled carbon network produced during in situ synthesis. Mechanical compression is another simple and practical method to reduce the surplus void space and increase the density of hybrid materials, but such a shrinkage from exterior to interior inevitably destroys the hybrid structure and is unfavorable to retain the stable electrode structure in discharge–charge process^28^.

Graphene, a two-dimensional and flexible carbon material, can provide an ultra-large contact area with noncarbons, which has enabled it to be used to build high-performance hybrid electrodes with the least carbon content^29^. The compact assembly of graphene, free of inter-particle voids, has shown the possibility for high volumetric energy storage^30,31^. As a typical example, a hybrid hydrogel of noncarbon and the three-dimensional (3D) graphene network was synthesized by a hydrothermal process, which was subsequently treated with a capillary evaporation drying to achieve a shrinkage starting in the middle of the sample, yielding a 3D ultrahigh density assembly. But it is difficult to ensure that, during this in situ shrinkage, enough void space is left for the noncarbon expansion. Consequently, as illustrated in Fig. 1a, this graphene cage with inadequate void space will crack during lithiation, leading to the pulverization of the noncarbons and rapid capacity fade. To yield an enough void space to buffer volume changes in carbon cages in the reported works, the template technique using removable templates of Si spheres^32^, nickel (Ni) foam^33^, polystyrene (PS) spheres^25^ and some salt templates^34,35^ is mainly used. However, these templates used are somewhat incompatible with the noncarbon particles, and usually create template shape-induced voids, making it difficult to precisely tune and distribute the void space even with complicated procedures.

In this work, we develop a strategy of sulfur-templated shrinkage to prepare graphene cages with a high-density but well-defined void space around noncarbon active materials. Typically, in the capillary drying of networked graphene hydrogels, flowable, deformable and removable sulfur is an ideal volume template leaving exact voids for the expansion of the noncarbon nanoparticles. In contrast to the above-mentioned hard templates, soft sulfur can encapsulate noncarbon particles even of nanometer size (<10 nm) without any gap between them. In hydrothermal process, sulfur, like Transformers presented in a famous film, possessing both fluidity and viscosity, covers every single noncarbon particle and therefore prevents noncarbon particles from aggregation, and the strong interaction between the sulfur and the noncarbon components ensures the tight contact between them. The sulfur is used as a void space precursor around noncarbons in a shrinking 3D graphene cage, and a precisely tuned and well-distributed void space at the nanoscale is guaranteed after sulfur removal (Fig. 1b). As a typical example, a nanosized tin oxide@graphene cage hybrid (SnO_2_@GC) with 67 wt% SnO_2_ is prepared, which is characterized by a high specific capacity (974 mAh g^–1^) and an ultrahigh volumetric capacity of 2123 mAh cm^–3^ due to the well-designed void (~260%) in a graphene cage for expansion of SnO_2_ upon lithiation.

## Results

### Synthesis and characterization of SnO2@GC

To obtain this hybrid, amorphous sulfur and SnO_2_ nanoparticles (SnO_2_ NPs) were embedded in a reduced graphene oxide hydrogel by a one-step hydrothermal method (Supplementary Fig. 1a, b). Capillary evaporation drying was then used to eliminate any voids, forming a compact graphene network. After sulfur removal, buffer space remains, producing a material that allows complete SnO_2_ expansion (Supplementary Fig. 2). The buffer space available is determined by simply controlling the amount of sulfur, and this is applicable for any other noncarbon anode materials.

Thermogravimetric analysis (TGA) under an inert atmosphere shows that the sulfur was almost completely removed by a mild thermal treatment of 400 ^o^C^36^(Supplementary Fig. 1c). The elemental analysis shows the residual sulfur is 0.87%, which may ascribe to the strong interaction between sulfur and SnO_2_ (Supplementary Fig. 3). In spite of the sulfur removal possibly being in the liquid state, the low surface tension between graphene and liquid sulfur did not lead to further shrinkage of the graphene cage (Supplementary Fig. 5 and Supplementary Fig. 6). Thus, the void volume is easily and precisely tuned by changing the sulfur content (Supplementary Fig. 1c and Supplementary Fig. 6b, c). The hybrid material is denoted SnO_2_@GC-*X*, where *X* (*X* = 0, 5, 11, 15, 21, 49) corresponds to the % original sulfur content that is all removed_._ The 3D stable structure after sulfur removal consists of mechanically robust graphene and is clearly characterized in Supplementary Fig. 4.

**Fig. 2:**
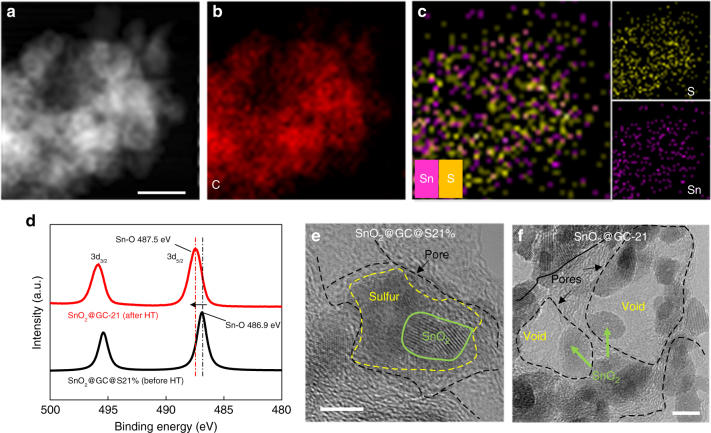
Characterization of the state of the sulfur and the SnO_2_ NPs in the 3D graphene cages. **a** STEM and **b**,** c** EDS of SnO_2_@GC@S21% before heat treatment (HT) showing the distribution of elemental C, S and Sn, especially the distribution of S and Sn. **d** Sn 3d XPS spectra with a 0.6 eV shift between SnO_2_@GC-21 (after HT) and SnO_2_@GC@S21% (before HT). **e** HRTEM image of SnO_2_@GC@S21%. **f** HRTEM image of SnO_2_@GC-21. Scale bars: **a** 100 nm; **e**, **f** 5 nm

The key to this method is that the sulfur flows around the SnO_2_ NPs and remains there during the preparation process. We investigated the SnO_2_ NPs and sulfur distribution by scanning transmission electron microscopy (STEM) and energy-dispersive X-ray spectroscopy (EDS) mapping (Fig. 2a–c and Supplementary Fig. 9a, b). The EDS maps of Sn and S elements clearly indicate their affinity, which is ascribed to the strong interaction between them (Supplementary Fig. 7). X-ray photoelectron spectroscopy (XPS) analysis was conducted to probe the chemical state of each element, and the spectra of C 1s indicates further reduction of graphene cage in thermal treatment (Supplementary Fig. 8). A shift of 0.6 eV in the Sn 3d XPS spectra appears for the Sn-O bond before and after sulfur removal (Fig. 2d), suggesting that sulfur removal leads to a chemical environment change around the SnO_2_ NPs^37,38^. Before sulfur removal, a high-resolution transmission electron microscopy (HRTEM) image of amorphous sulfur and the distribution of SnO_2_ NPs (Fig. 2e) shows the sulfur encapsulation for SnO_2_ NP in the individual pore. After sulfur removal, the ultrafine SnO_2_ NPs (sizes of 5–10 nm) are clearly seen with a uniform distribution of void space around them (Fig. 2f). The lattice resolution images (Supplementary Fig. 10) clearly show the characteristic lattice fringe of 0.33 nm corresponding to the (110) plane of SnO_2_^39^. The introduction of sulfur prevents the aggregation of SnO_2_ NPs, retaining a high degree of dispersion. Agglomeration occurs only in the sample without sulfur (Supplementary Fig. 9c, d). The removal of excess sulfur produces larger gaps between the SnO_2_ NPs, meaning increased void space around them (Supplementary Fig. 9e–h). For comparison, typical hard templates of PS spheres and sodium chloride (NaCl) were used to incorporate void space for the SnO_2_ NPs in the graphene cages. However, due to undesirable contact with the SnO_2_ NPs, removal of the PS spheres failed to provide distributed voids for the SnO_2_ NPs (Supplementary Fig. 11) and NaCl destroyed the stable 3D graphene structure during capillary drying, and did not provide sufficient voids for most of the SnO_2_ NPs (Supplementary Fig. 12).

**Fig. 3:**
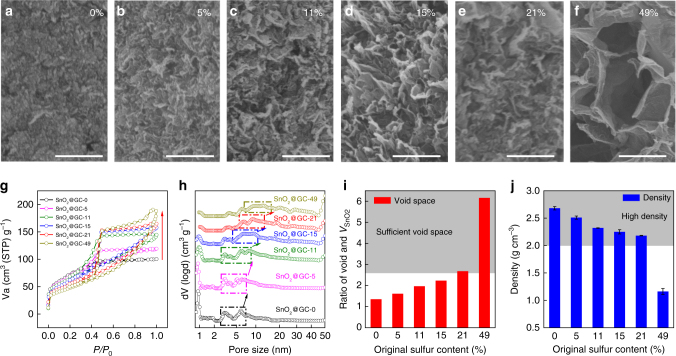
Tuning the void space of SnO_2_@GCs at the nanoscale. **a**–**f** SEM images showing the more expanded structure of SnO_2_@GCs containing increasing amounts of sulfur (0, 5, 11, 15, 21, 49%) after it has all been removed. **g** Nitrogen adsorption–desorption isotherms, and **h** pore size distributions of SnO_2_@GCs showing that the pore structure changes and pore size increases as the original sulfur content increases. **i** Calculated void space and **j** density changes as a result of removing the sulfur from the six samples. Error bars indicate s.d. (*n* = 3). Scale bars: **a**–**f** 500 nm

The influence of sulfur content on the void space has been clearly demonstrated by scanning electron microscopy (SEM) in Fig. 3a–f. As shown in Fig. 3a, without sulfur, after capillary evaporation the 3D graphene assembly shrinks to a condensed and compact solid with no apparent pores. When sulfur is present, the graphene network shrinkage was resisted, and a larger monolith resulted from more sulfur (Supplementary Fig. 6b). As shown in Fig. 3b–f, with more sulfur present in the original, more open and expanded pores were left. Details of the void space changes in the SnO_2_@GCs can be seen from the nitrogen (N_2_) adsorption–desorption isotherms (Fig. 3g). As the amount of sulfur in the original material increased, the obtained SnO_2_@GCs showed decreased adsorption in the middle relative pressure range but a larger hysteresis loop, suggesting that the sulfur mainly creates large mesopores, consistent with the TEM images (Fig. 2f). The pore size distributions (Fig. 3h) confirm that the pore size gradually increased from 3 nm to 15 nm as the original sulfur content increased. Note that the micropores almost disappear, suggesting that the sulfur effectively prevents the shrinkage of the graphene network. The increased number of large mesopores and macropores leads to a larger pore volume (Supplementary Fig. 13 and Supplementary Fig. 14). In SnO_2_@GC-0, 5, 11, 15, 21 and 49, the original sulfur volumes are respectively 0, 0.26, 0.62, 0.88, 1.33 and 4.81 times the SnO_2_ volume. Adding this extra volume generated by the sacrificial sulfur, the corresponding total calculated void spaces are around 1.35, 1.61, 1.97, 2.23, 2.68 and 6.16 times larger than the SnO_2_ volume^28^ (Fig. 3i). Note that the void space of SnO_2_@GC-21 exactly satisfies the space requirement for the full expansion of SnO_2_^39,40^. These results confirm that the required void space can be precisely incorporated by controlling the amount of sulfur used. Although larger void space leads to a lower density, SnO_2_@GC-21 still has a high bulk density of 2.18 g cm^–3^ (Fig. 3j).

### Electrochemical performance and in situ TEM characterization

**Fig. 4:**
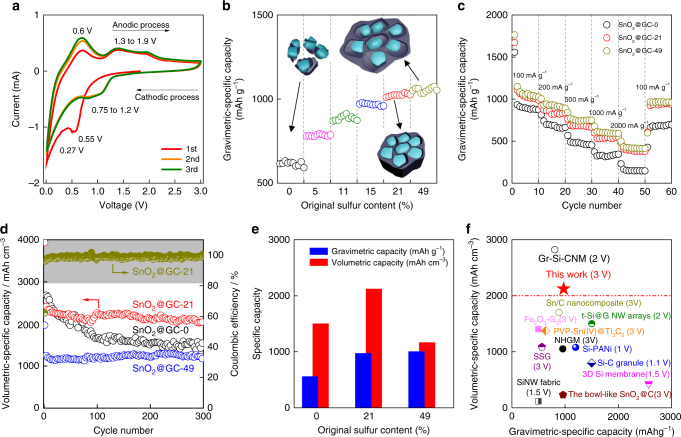
Electrochemical characterization of SnO_2_@GCs. **a** Representative cyclic voltammetry curves (CVs) of SnO_2_@GC-21 at a scan rate of 0.5 mV s^–1^. **b** The gradient electrochemical performance change of SnO_2_@GCs based on the different original sulfur contents when the gravimetric capacity became stable at around the 130^th^–140^th^ cycles (100 mA g^–1^). The overall cycling performance of SnO_2_@GCs can be observed in Supplementary Fig. 17. **c** Rate performance of SnO_2_@GCs. **d** Cycling performance of SnO_2_@GC-0, 21 and 49 at a current density of 100 mA g^–1^. **e**, **f** Comparison of the volumetric and gravimetric specific capacities of SnO_2_@GC-21 with the referenced cases (based on the total active materials). **e** Comparison with SnO_2_@GC-0 and SnO_2_@GC-49. **f** Comparison with other reported anode materials (based on the total active materials), such as t-Si@G NW arrays^5^, Fe_2_O_3_-G_2_^6^, PVP-Sn(IV)@Ti_2_C^7^_,_ Sn/C nanocomposite^8^, SSG^9^, Si-C granule^10^_,_ Si-PANi^13^, NHGM^15^, SiNW fabric^22^, 3D Si membrane^23^ and the bowl-like SnO_2_@C^27^, and Gr-Si-CNM^45^, with a voltage it charged to (see details in Supplementary Table 1)

To evaluate the electrochemical properties, coin-type cells were assembled with lithium (Li) as the counter electrode. Figure 4a shows the cyclic voltammetry (CV) curves for the first three cycles of SnO_2_@GC-21 in the range of 0.01 to 3 V, at a scan rate of 0.5 mV s^–1^. The peak shift from the first cycle to subsequent cycles is mainly the result of the SEI formation and partial irreversibility of lithium oxide (Li_2_O) formation^41^. The irreversible capacity loss can be reduced by decreasing the specific surface area of graphene network, and downsizing the SnO_2_ NP to improve the reversibility of Li_2_O.

As shown in Fig. 4b, although the initial discharge gravimetric-specific capacities of all samples are similar (Supplementary Fig. 15a), SnO_2_@GC-0 shows large capacity decay due to the lack of expansion volume (Supplementary Fig. 16). If not specifically mentioned, the gravimetric capacities are based on the total mass of SnO_2_@GC (including both SnO_2_ and graphene). SnO_2_@GC-0 has a gravimetric-specific capacity of only 592 mAh g^–1^ after 130 cycles. Interestingly, with the increase of the volume for expansion, the gravimetric-specific capacity also gradually increases. When the original sulfur content reaches 21%, the gravimetric-specific capacity of SnO_2_@GC-21 reaches a peak value of 1025 mAh g^–1^ with the same cycling numbers as SnO_2_@GC-0. Therefore, an original sulfur content of 21% should create the smallest void space that is needed for lithiation, which is in agreement with the earlier calculation. The initial charge and discharge capacities of SnO_2_@GC-21 are 1121 mAh g^–1^ and 1805 mAh g^–1^, corresponding to a high Coulombic efficiency of 62.1% (Supplementary Fig. 15b). After 300 cycles under a current density of 100 mA g^–1^, both the discharge and charge capacities of this material are stable at 974 mAh g^–1^, delivering 84% capacity retention, which is better than that of other SnO_2_@GCs with fewer voids (Supplementary Fig. 17). It is noted that with enough void space, good rate performance has been obtained, with gravimetric-specific capacity of 599 mAh g^–1^ at 1000 mA g^–1^, and 476 mAh g^–1^ at 2000 mAh g^–1^ (Fig. 4c). Compared with SnO_2_@GC-21, SnO_2_@GC-0 with insufficient void space has an inferior gravimetric capacity after cycling in varied rates (Supplementary Fig. 18). Thus, the excellent cycling stability of SnO_2_@GC-21 can be mainly ascribed to the sufficient void space for Li-ion diffusion and the SnO_2_ volume change. In this work, the gravimetric capacity of SnO_2_@GC-21 can be further improved by lowering the SnO_2_ NP size and optimizing the interface between the graphene and SnO_2_ NP^42–44^.

A more attractive result is that the SnO_2_@GC-21 has a superior volumetric capacity. The density of SnO_2_@GC-21 is up to 2.18 g cm^–3^ and its volumetric-specific capacity can reach 2415 mAh cm^–3^ at a current density of 100 mA g^–1^ and even 1036 mAh cm^–3^ with a current density of 2000 mA g^–1^. The volumetric capacity calculation considers the total volume of SnO_2_ and graphene in SnO_2_@GC hybrid. The volumetric-specific capacity of 2523 mAh cm^–3^ in the initial reversible cycle is obtained (Fig. 4d), and after 300 cycles, the ultrahigh volumetric capacity of 2123 mAh cm^–3^ is still retained, which far exceeds the SnO_2_@GC-0 and SnO_2_@GC-49 (Fig. 4e). To the best of our knowledge, this is among the highest of volumetric-specific capacities based on the active material among all Sn-based and Si-based hybrid active materials^45^ (Fig. 4f and Supplementary Table 1). This traditional slurry casting electrode also offers a high volumetric capacity of 1075 mAh cm^–3^ with the volume addition of polyvinylidene fluoride (PVDF) binder and carbon black, which is comparable to the record high volumetric value of Si-based electrodes (Supplementary Fig. 19 and Supplementary Table 1). The dense and thick SnO_2_@GC-21 electrode with active material mass loading up to 3.5 mg cm^–2^ was also tested, and the reversible areal capacity reaches 3.3 mAh cm^–2^ with a good cyclability (Supplementary Fig. 20). A full-cell test using electrochemical pre-lithiation technology was further performed to recognize the potential of SnO_2_@GC-21 towards the practical application. The galvanostatic cycling of a full cell using lithium cobalt oxide (LCO) as cathode with an operating voltage of 2.6–4.35 V shows a high first-cycle Coulombic efficiency of 91%, which is comparable to that of commercial graphite anode. The LCO/SnO_2_@GC-21 full cell also shows a high Coulombic efficiency over only initial 4 cycles and stable cycling at gravimetric capacity of 135 mAh g^–1^ based on LCO (Supplementary Fig. 21).

To further prove the above conclusion, the influence of SnO_2_ content on the electrochemical performance was also investigated. Supplementary Figure 22 shows the denser structure, smaller pore size and lower total pore volume with the increase of SnO_2_ loading. Subsequently, SnO_2_@GC-SnO_2_0%, 46%, 67% and 75% with same original sulfur loading (Supplementary Fig. 1d) were cycled at a current density of 100 mA g^–1^ (Supplementary Fig. 23). After 100 cycles, the SnO_2_@GC-SnO_2_67% with 21% original sulfur content (SnO_2_@GC-21) still delivers the highest volumetric specific capacitance. Although SnO_2_@GC-SnO_2_75% is more compact, too much SnO_2_ reduces the conductivity and cannot produce enough expansion volume.

As shown in Supplementary Fig. 24, Nyquist plots show that the diameters of the semicircles for SnO_2_@GC-11, 21 and 49 in the high–medium frequency region are much smaller than that of SnO_2_@GC-0, indicating the greatly decreased charge-transfer resistance because of the tight and uniform contact of the graphene with the SnO_2_ NPs. Additionally, the Warburg segment length of SnO_2_@GC-21, 49 is much shorter due to sufficient void space favoring Li-ion diffusion, which guarantees a superior rate performance. Thus, we can conclude that the high capacity, excellent cycling stability and rate capability of SnO_2_@GC-21 are due to its unique hybrid structure.

**Fig. 5:**
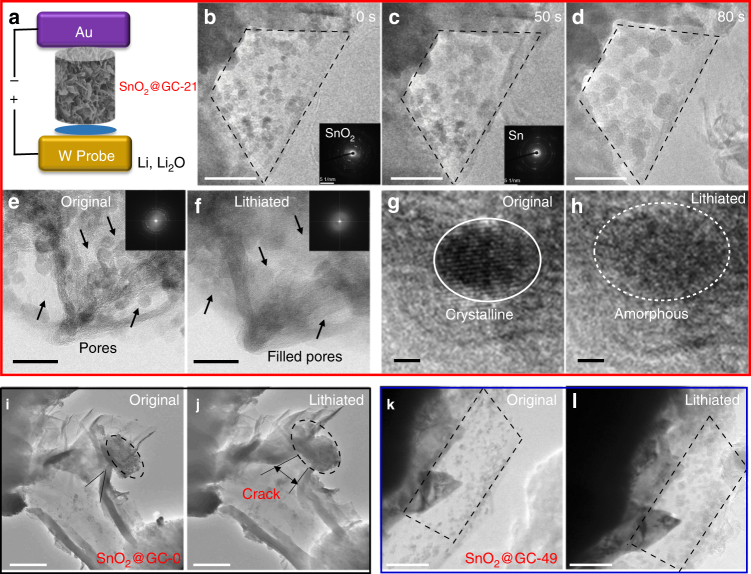
In situ TEM probing of the SnO_2_ NP expansion in a graphene cage. **a** Schematic and captured in situ TEM images from time-lapse movies of **b**–**h** SnO_2_@GC-21 (Supplementary Movie 1–3), **i**,** j** SnO_2_@GC-0 (Supplementary Movie 4) and **k**,** l** SnO_2_@GC-49 (Supplementary Movie 5). The lithiation process within graphene encapsulation is obviously interpreted by in situ TEM images. Graphene cage fracture occurred when aggregated SnO_2_ NPs in SnO_2_@GC-0 completely expanded, while the SnO_2_@GC-21, 49 samples have enough space for lithium storage, especially the SnO_2_@GC-21 whose void space is fully utilized by the expansion. Scale bars: **b**–**d** 50 nm; **e**,** f** 20 nm; **g**, **h** 2 nm; **i**, **j** 200 nm; **k**, **l** 100 nm

To better analyze the whole lithium storage process of SnO_2_ NPs, in situ and ex situ TEM electrochemical tests were carried out. As shown in Fig. 5a, the electrochemical micro-cell consists of a gold (Au) rod decorated with the SnO_2_@GC as the working electrode and a tungsten (W) probe with a small piece of Li covered with a layer of Li_2_O attached to its tip as the counter electrode. A series of TEM images for SnO_2_@GCs was recorded during the lithiation process (Supplementary movies 1–5). The results show that, after the contact of the two electrodes, the initial particles start to expand due to the lithiation. In situ and ex situ TEM images (Fig. 5b–d, Supplementary Movie 1 and Supplementary Fig. 25a) clearly show that the SnO_2_ NPs in SnO_2_@GC-21 gradually expand to fill the gaps between the SnO_2_ NPs, indicating that all the void space is used during the lithiation process. Figure 5e, f in a higher resolution demonstrate that the expansion of SnO_2_ NPs occurs within the pores of graphene network and the graphene cage has almost no volume change following lithiation, confirming the existence of enough internal void space in SnO_2_@GC-21 to buffer the complete expansion of SnO_2_ NPs (Supplementary Movie 2). A selected area electron diffraction indicates the production of Sn during lithiation and the amorphous structure formation of the Li–Sn alloy after complete lithiation at 0.01 V (Fig. 5c and Supplementary Fig. 25a–c), which is also characterized by the fast Fourier transformation images in Fig. 5e, f. Moreover, the high-resolution in situ TEM images clearly show the transformation from the individual SnO_2_ NP into the amorphous Li–Sn alloy upon lithiation (Fig. 5g, h and Supplementary Movie 3). Insufficient void space for SnO_2_ expansion leads to severe structural fracture that can be observed in the lithiation of SnO_2_@GC-0 (Fig. 5i, j, Supplementary Movie 4). From the ex situ TEM image of SnO_2_@GC-0, some SnO_2_ NPs are not lithiated at 0.01 V, which is possibly due to difficult ion diffusion in such a highly compact structure (Supplementary Fig. 25d–f). For SnO_2_@GC-49 with excess void space, there is space remaining even after the full expansion of the SnO_2_ NPs, which leads to poor space usage (Fig. 5k, l, Supplementary Movie 5 and Supplementary Fig. 25g).

### Generalization to other noncarbon materials

To show the generality of the sulfur template method for other high volumetric capacity anode, we also prepared silicon@graphene cage hybrid (Si@GC). The presence of Si and the content of sulfur and Si are confirmed by the X-ray diffraction (XRD) and TGA results, respectively (Supplementary Fig. 26). Supplementary Figure 27 shows that sulfur encapsulates Si NPs due to the affinity between sulfur and Si^46^. Also, the Si NPs (~50 nm) are well caged by 3D graphene network (Supplementary Fig. 28a). SEM images (Supplementary Fig. 28b–d), N_2_ adsorption–desorption isotherms (Supplementary Fig. 28e) and pore size distributions (Supplementary Fig. 28f) show that the sulfur around the Si NPs is all removed, leaving larger void space for the volume expansion of the Si NPs with higher sulfur content, which indicates the generality of the method for other noncarbon particles, even with a larger size. As a result of the precise void space control provided by a sulfur template, the gravimetric performance could be optimized in a relatively compact graphene cage of Si@GC-44 with sufficient void space (3.67 times than the Si volume), also with a high density of 0.79 g cm^–3^ (Supplementary Fig. 28g, h), achieving a high volumetric capacity of 773 mAh cm^–3^ with good cyclic stability at 200 mA g^–1^ (Supplementary Fig. 29).

## Discussion

To achieve an ultrahigh volumetric capacity, we have presented an effective approach using flowable, deformable and removable sulfur as a template to precisely control the void space around SnO_2_ nanoparticles, including both its size and location in a shrinking 3D graphene cage. Our material design fulfills the most stringent requirements for balancing the complete expansion of SnO_2_ and the high density of the SnO_2_@GC hybrids. Ultrahigh volumetric capacities of 2123 mAh cm^–3^ and 1075 mAh cm^–3^, respectively, for the SnO_2_@GC (active materials only) and the whole electrode with good cyclic stability are achieved. Since graphenes are seen as the basic units for all *sp*^2^ carbons, this study of graphene-assembled carbons represents a perfect design for carbon cages housing nanocarbon electrodes in lithium-ion batteries. Also, this strategy has proved its generalization to other noncarbon anodes for lithium-ion batteries to buffer large volume expansions during electrochemical reactions and is absolutely an ideal remedy for low volumetric energy density in energy storage devices with carbon cages as electrochemical reaction frameworks, not just limited to lithium-ion batteries.

## Methods

### Fabrication of SnO_2_@GC

Graphite oxide (GO) powder was fabricated using a modified Hummers method. Different amounts of sodium thiosulfate powder (Na_2_S_2_O_3_·5H_2_O) were added to 57 mL of the GO (2 mg mL^–1^) suspension and stirred for 1 h followed by the dropwise addition of hydrochloric acid (HCl, 1 mol L^–1^) to completely react with the Na_2_S_2_O_3_·5H_2_O to produce sulfur. Subsequently, a certain amount of tin chloride pentahydrate (SnCl_4_·5H_2_O) as the precursor of SnO_2_ was added to the GO and sulfur suspension and strongly stirred for 1 h. The prepared solution was sealed in a 100 mL Teflon-lined autoclave and hydrothermally treated at 180 ^o^C for 6 h to obtain a cylindrical SnO_2_@graphene@sulfur hybrid hydrogel. This hydrogel was washed to remove excess Na^+^, Sn^4+^ and Cl^–^, then subjected to evaporation-induced drying for 48 h, followed by thermal treatment at 400 ^o^C of 6 h. For reference, a SnO_2_@graphene macroform without sulfur was prepared using the same thermal treatment. The preparation of Si@GC was similar to that of the SnO_2_@GC with the use of a sulfur template. PS spheres were used and removed using the same procedures as for the sulfur template, while the use of a NaCl template meant that the SnO_2_@GC hydrogel was soaked in a 50 mL NaCl solution (4 M) for static adsorption of 12 h and subsequently dried with the capillary evaporation approach. The SnO_2_@GC@NaCl was repeatedly washed by de-ionized water to obtain SnO_2_@GC. The main experimental parameters are shown in Supplementary Table 2.

### Material characterizations

Phase purity and crystal structure were characterized by XRD (Bruker D-8 diffractometer, Cu Kα radiation, *λ = *0.154 nm). Thermogravimetric analysis (TG, Rigaku, Japan) was performed to calculate the sulfur, SnO_2_ and Si contents. SEM and TEM observations were performed on a Hitachi S-4800 (Hitachi, Japan) and a JEM 2100F (JEOL, Japan), respectively; EDS was used for the elemental analysis. N_2_ adsorption–desorption was measured using a BEL mini-instrument, and specific surface areas and pore size distribution were obtained using the Brunauer–Emmett–Teller (BET) and density function theories methods, respectively. XPS analyses were conducted with a Physical Electronics PHI5802 instrument using a magnesium anode (monochromatic Kα X-rays at 1253.6 eV) as the source. Raman spectra were recorded using a multi-wavelength micro-Raman spectroscope (JY HR800) using 532 nm incident radiation and a 50× aperture. The Mercury intrusion porosimetry was conducted with an AutoPore IV 9500. The elemental composition characterization was performed on an element analyzer of Vario MACRO cube.

### Void space calculation

Considering the void volume originally occupied by sulfur in SnO_2_@GC-0, 5, 11, 15, 21 and 49, the volume ratio of the sulfur volume and the SnO_2_ volume can be calculated as:

1 $${\it{T}}_{\mathrm{s}} = \frac{{{\it{V}}_{{\rm sulfur}}}}{{{\it{V}}_{{\rm SnO_2}}}} = \frac{{{\it{\omega }}_{{\mathrm{ }}{\rm sulfur}}^\# }}{{{\it{\rho }}_{{\mathrm{ }}{\rm sulfur}}}}/\frac{{{\it{\omega }}_{{\mathrm{ }}{\rm SnO_2}}^\# }}{{{\it{\rho }}_{{\mathrm{ }}{\rm SnO_2}}}},$$

where ${\it{\omega }}_{{\mathrm{ }}{\rm sulfur}}^\#$ and ${\it{\omega }}_{{\mathrm{ }}{\rm SnO_2}}^\#$ are respectively the sulfur content and SnO_2_ content based on the whole SnO_2_@GC@S macroform. In particular, the SnO_2_ content based on the whole SnO_2_@GC@S macroform is calculated by ${\it{\omega }}_{{\mathrm{ }}{\rm SnO_2}}^\# {\mathrm{ = }}{\it{\omega }}_{{\mathrm{ }}{\rm SnO_2}} \times (1 - {\it{\omega }}_{{\mathrm{ }}{\rm sulfur}}^\# )$, and ${\it{\omega }}_{{\mathrm{ }}{\rm SnO_2}}$ is the SnO_2_ content in SnO_2_@GC, which is 67%. ${\it{\rho }}_{{\mathrm{ }}{\rm sulfur}}$ and ${\it{\rho }}_{{\mathrm{ }}{\rm SnO_2}}$ are the densities of sulfur and SnO_2_, which are respectively 2.07 g cm^–3^ and 6.95 g cm^–3^. Thus, ${\it{T}}_{\mathrm{s}}$ is the volume ratio of the original sulfur volume to the total SnO_2_ volume, and in SnO_2_@GC-0, 5, 11, 15, 21 and 49, the values are around 0, 0.26, 0.62, 0.88, 1.33 and 4.81.

The volume ratio of the void space in SnO_2_@GC-0 and the total SnO_2_ volume can be calculated as:

2 $$6.95 \times {\it{V}}_{\rm {Sn}O_2} + 2.25 \times {\it{V}}_{\rm C} = 1,$$

3 $$\frac{{6.95 \times {\it{V}}_{\rm {Sn}O_2}}}{{2.25 \times {\it{V}}_{\rm C}}} = \frac{{0.67}}{{0.33}},$$

4 $$\frac{1}{{{\it{V}}_{{\rm {Sn}O_2}}{\mathrm{ + }}{\it{V}}_{\rm C}{\mathrm{ + }}{\it{V}}}}{\mathrm{ = }}{\it{\rho }},$$

where 2.25 is the density of graphite; ${\it{V}}_{\rm {Sn}O_2}$ and ${\it{V}}_{\rm C}$ are respectively the SnO_2_ and graphene volumes in SnO_2_@GC and ${\it{V}}$ is the void space in SnO_2_@GC-0; ${\it{\rho }}$ is the density of SnO_2_@GC-0, which is 2.68 g cm^–3^. According to Eqs. 2–4, the volume ratio of the void space in SnO_2_@GC-0 to the total SnO_2_ volume is 1.35. There is no sulfur for void incorporation in SnO_2_@GC-0, and thus adding the extra volume generated by sacrificial sulfur in SnO_2_@GC-0, 5, 11, 15, 21 and 49, the corresponding total calculated void volumes are around 1.35, 1.61, 1.97, 2.23, 2.68 and 6.16 times larger than the SnO_2_ volume. The detailed results of the calculated void space are presented in Supplementary Table 3.

### Electrochemical measurements

For half cells, the battery performance was evaluated by the galvanostatic cycling of coin cells with the SnO_2_@GC or the Si@GC as the working electrode and Li foil as the counter electrode with a porous polypropylene film as the separator, and an electrolyte of 1M lithium hexafluorophosphate (LiPF_6_) in 1:1 (v/v) ethylene carbonate/diethyl carbonate. The working electrode was made using the typical slurry method with 80 wt% SnO_2_@GC, 10 wt% carbon black and 10 wt% PVDF in *N*-methyl-2-pyrrolidone, which were stirred for 5 h and then coated onto a Cu foil and vacuum-dried at 50 ^o^C for 24 h. The foil was then cut into a circular pallet with a diameter of 12 mm and used as the anode. A 2032 coin cell was assembled in an Ar-filled glovebox (MBraum), and to use as a test cell that was examined on the battery testers (LAND, China). Electrochemical impedance spectra and CV characterizations were conducted by the electrochemistry workstations (Metrohm, Switzerland). For full cell, the SnO_2_@GC was paired with a LCO cathode with an N/P ratio of ~1.1.

If not specifically mentioned, all the gravimetric capacities are calculated based on the mass of SnO_2_@GC.

The volumetric-specific capacity was calculated as:

5 $${\it{C}}_{\rm v} = {\it{C}}_{\mathrm{g}} \times {\it{\rho }},$$

where ${\it{C}}_{\mathrm{v}}$ is the volumetric-specific capacity, mAh cm^–3^; ${\it{C}}_{\mathrm{g}}$ is the gravimetric-specific capacity, mAh g^–1^; ${\it{\rho }}$ is the density of active material or electrode, g cm^–3^_._

The volumetric-specific capacity based on the active material (${\it{C}}_{{\mathrm{v}},1}$) was calculated as:

6 $${\it{C}}_{{\mathrm{v}},1} = {\it{C}}_{{\mathrm{g,1}}} \times {\it{\rho }}_{{\mathrm{ }}1},$$

where ${\it{C}}_{{\rm v},1}$ is the volumetric-specific capacity of active material, mAh cm^–3^; ${\it{C}}_{{\mathrm{g,1}}}$ is the gravimetric-specific capacity based on the total amount of active material including both SnO_2_ and graphene, mAh g^–1^. The density of the active material (SnO_2_@GC) is the monolith density determined by Archimedes principle with a balance (Mettler Toledo XS205) equipped with accessories.

The volumetric-specific capacity based on whole electrode (${\it{C}}_{{\mathrm{v}},2}$) was calculated as:

7 $${\it{C}}_{{\mathrm{v}},2} = {\it{C}}_{{\mathrm{g,2}}} \times {\it{\rho }}_{{\mathrm{ 2}}},$$

where ${\it{C}}_{{\mathrm{v}},2}$ is the volumetric-specific capacity based on the electrode, mAh cm^–3^; ${\it{C}}_{{\mathrm{g,2}}}$ is the gravimetric-specific capacity based on the whole electrode weight (SnO_2_@GC, PVDF binder and carbon black. The weight fraction of the active material in electrode is 0.8, thus, ${\it{C}}_{{\rm g},2} = {\it{C}}_{{\mathrm{g,1}}} \times 0.8$), mAh g^–1^; ${\it{\rho }}_{{\mathrm{ 2}}}$ is the density of electrode based on the whole volume of SnO_2_@GC, PVDF binder and carbon black, g cm^–3^.

### In situ TEM characterization

In situ TEM images were taken on a JEOL-3100 FEF equipped with an Omega filter and a Nanofactory Instruments STM-TEM holder. In order to build the test cell, SnO_2_@GC powders were attached to the gold rod, which was further attached to the piezo-manipulator. A small piece of Li foil covered with a Li_2_O layer was attached to a tungsten probe as the counter electrode. During our experiments, the SnO_2_@GC was loaded onto the edge of a gold rod with a freshly cut tip by simply scratching the rod against bulk powder of SnO_2_@GC. The opposite tip coated with Li_2_O and Li was in the micrometer scale. The lithiation was carried out at a negative bias in the range of −2 V with respect to the Li metal.

### Data availability

The data supporting the findings of this work are available within the article and its Supplementary Information files. All other relevant data supporting the findings of this study are available from the corresponding author on request.

## Electronic supplementary material

Supplementary Information

Description of Additional Supplementary Files

Supplementary Movie 1

Supplementary Movie 2

Supplementary Movie 3

Supplementary Movie 4

Supplementary Movie 5

## References

[1] Gogotsi Y, Simon P (2011). True performance metrics in electrochemical energy storage. Science.

[2] Zhang C, Lv W, Tao Y, Yang QH (2015). Towards superior volumetric performance: design and preparation of novel carbon materials for energy storage. Energy Environ. Sci..

[3] Goodenough JB, Kim Y (2010). Challenges for rechargeable Li batteries. Chem. Mater..

[4] Kovalenko I (2011). A major constituent of brown algae for use in high-capacity Li-ion batteries. Science.

[5] Wang B (2013). High volumetric capacity silicon-based lithium battery anodes by nanoscale system engineering. Nano Lett..

[6] Li Z (2017). Twin-functional graphene oxide: compacting with Fe_2_O_3_ into a high volumetric capacity anode for lithium ion battery. Energy Storage Mater..

[7] Luo JM (2016). Sn^4+^ ion decorated highly conductive Ti_3_C_2_ Mxene: promising lithium-ion anodes with enhanced volumetric capacity and cyclic performance. ACS Nano.

[8] Liu JY (2016). High volumetric capacity three-dimensionally sphere-caged secondary battery anodes. Nano Lett..

[9] Yin JF, Cao HQ, Zhou ZF, Zhang JX, Qu MZ (2012). SnS_2_@reduced graphene oxide nanocomposites as anode materials with high capacity for rechargeable lithium ion batteries. J. Mater. Chem..

[10] Magasinski A (2010). High-performance lithium-ion anodes using a hierarchical bottom-up approach. Nat. Mater..

[11] Son IH (2015). Silicon carbide-free graphene growth on silicon for lithium-ion battery with high volumetric energy density. Nat. Commun..

[12] Li YZ (2016). Growth of conformal graphene cages on micrometre-sized silicon particles as stable battery anodes. Nat. Energy.

[13] Wu H (2013). Stable Li-ion battery anodes by *in-situ* polymerization of conducting hydrogel to conformally coat silicon nanoparticles. Nat. Commun..

[14] Pumera M (2011). Graphene-based nanomaterials for energy storage. Energy Environ. Sci..

[15] Wang X (2016). High-density monolith of N-doped holey graphene for ultrahigh volumetric capacity of Li-ion batteries. Adv. Energy Mater..

[16] Su FY (2010). Flexible and planar graphene conductive additives for lithium-ion batteries. J. Mater. Chem..

[17] Lv W, Li Z, Deng Y, Yang QH, Kang F (2016). Graphene-based materials for electrochemical energy storage devices: opportunities and challenges. Energy Storage Mater..

[18] Liu N (2014). A pomegranate-inspired nanoscale design for large-volume-change lithium battery anodes. Nat. Nanotechnol..

[19] Zhang L, Wu HB, Madhavi S, Hng HH, Lou XW (2012). Formation of Fe_2_O_3_ microboxes with hierarchical shell structures from metal-organic frameworks and their lithium storage properties. J. Am. Chem. Soc..

[20] Tan GQ (2016). Freestanding three-dimensional core-shell nanoarrays for lithium-ion battery anodes. Nat. Commun..

[21] Yang SB, Feng XL, Mullen K (2011). Sandwich-like, graphene-based titania nanosheets with high surface area for fast lithium storage. Adv. Mater..

[22] Chockla AM (2011). Silicon nanowire fabric as a lithium ion battery electrode material. J. Am. Chem. Soc..

[23] Xia F (2013). Facile synthesis of free-standing silicon membranes with three-dimensional nanoarchitecture for anodes of lithium ion batteries. Nano Lett..

[24] Sun YM, Liu NA, Cui Y (2016). Promises and challenges of nanomaterials for lithium-based rechargeable batteries. Nat. Energy.

[25] Wu C, Maier J, Yu Y (2015). Sn-based nanoparticles encapsulated in a porous 3D graphene network: advanced anodes for high-rate and long life Li-ion batteries. Adv. Funct. Mater..

[26] Jahel A, Ghimbeu CM, Monconduit L, Vix-Guterl C (2014). Confined ultrasmall SnO_2_ particles in micro/mesoporous carbon as an extremely long cycle-life anode material for Li-ion batteries. Adv. Energy Mater..

[27] Liang J (2014). Bowl-like SnO_2_@carbon hollow particles as an advanced anode material for lithium-ion batteries. Angew. Chem. Int. Ed..

[28] Jin Y (2017). Self-healing SEI enables full-cell cycling of a silicon-majority anode with a coulombic efficiency exceeding 99.9%. Energy Environ. Sci..

[29] Tang R (2016). How a very trace amount of graphene additive works for constructing an efficient conductive network in LiCoO_2_-based lithium-ion batteries. Carbon N. Y..

[30] Tao Y (2013). Towards ultrahigh volumetric capacitance: graphene derived highly dense but porous carbons for supercapacitors. Sci. Rep..

[31] Yang XW, Cheng C, Wang YF, Qiu L, Li D (2013). Liquid-mediated dense integration of graphene materials for compact capacitive energy storage. Science.

[32] Schuster J (2012). Spherical ordered mesoporous carbon nanoparticles with high porosity for lithium-sulfur batteries. Angew. Chem. Int. Ed..

[33] Mo RW, Rooney D, Sun KN, Yang HY (2017). 3D nitrogen-doped graphene foam with encapsulated germanium/nitrogen-doped graphene yolk-shell nanoarchitecture for high-performance flexible Li-ion battery. Nat. Commun..

[34] Zhao H (2016). A convenient and versatile method to control the electrode microstructure toward high-energy lithium-ion batteries. Nano Lett..

[35] Li G (2016). Three-dimensional porous carbon composites containing high sulfur nanoparticle content for high-performance lithium-sulfur batteries. Nat. Commun..

[36] Zhang C (2015). A high-density graphene-sulfur assembly: a promising cathode for compact Li-S batteries. Nanoscale.

[37] Tian R (2016). The effect of annealing on a 3D SnO_2_/graphene foam as an advanced lithium-ion battery anode. Sci. Rep..

[38] Liu J (2016). SnO_2_ as a high-efficiency polysulfide trap in lithium-sulfur batteries. Nanoscale.

[39] Zhao KN (2016). SnO_2_ quantum dots@graphene oxide as a high-rate and long-life anode material for lithium-ion batteries. Small.

[40] Chiang YM (2010). Building a better battery. Science.

[41] Hu RZ (2016). Dramatically enhanced reversibility of Li_2_O in SnO_2_-based electrodes: the effect of nanostructure on high initial reversible capacity. Energy Environ. Sci..

[42] Zhou X, Wan LJ, Guo YG (2013). Binding SnO_2_ nanocrystals in nitrogen-doped graphene sheets as anode materials for lithium-ion batteries. Adv. Mater..

[43] Wang DN (2013). Layer by layer assembly of sandwiched graphene/SnO_2_ nanorod/carbon nanostructures with ultrahigh lithium ion storage properties. Energy Environ. Sci..

[44] Li XL (2016). Ultrafine SnO_2_ nanoparticles encased in graphene oxide nanoribbons for high-performance lithium ion batteries. Electrochim. Acta.

[45] Suresh S (2017). Protecting silicon film anodes in lithium-ion batteries using an atomically thin graphene drape. ACS Nano.

[46] Hassan FM (2015). Evidence of covalent synergy in silicon-sulfur-graphene yielding highly efficient and long-life lithium-ion batteries. Nat. Commun..

