# Supplementary material for: Caging tin oxide in three-dimensional graphene networks for superior volumetric lithium storage
Source: Nat Commun. 2018 Jan 26;9:402. doi: 10.1038/s41467-017-02808-2 (PMC5786064; doi:10.1038/s41467-017-02808-2)
Supplement: Supplementary file 1 — Supplementary Information [file 41467_2017_2808_MOESM1_ESM.pdf]

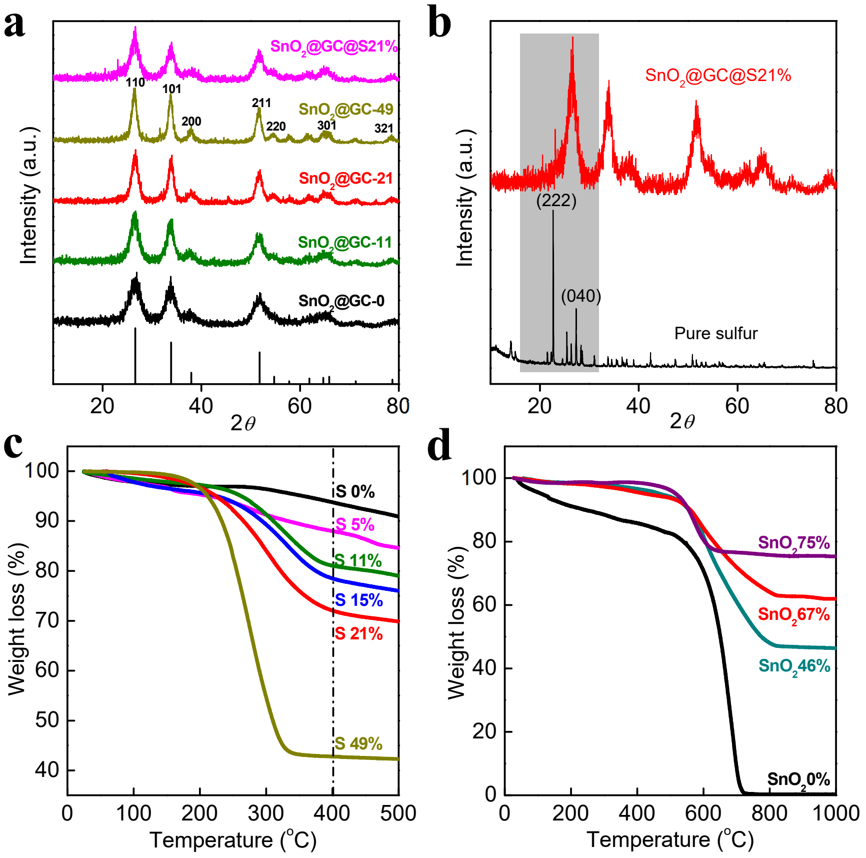

Supplementary Figure 1. The existence and content of SnO_2 in SnO_2@GCs. XRD patterns of **(a)** SnO_2@GC-0, 11, 21, 49, SnO_2@GC@S21% and **(b)** pure sulfur. **(c)** TGA curves of SnO_2@GCs with different original sulfur content of 0%, 5%, 11%, 15%, 21% and 49% in N_2 atmosphere. **(d)** TGA curves of SnO_2@GCs with the SnO_2 content of 0%, 46%, 67% and 75% measured in air atmosphere. Note that the sulfur content is calculated based on the whole SnO_2@GC@S, while the SnO_2 content is calculated based on the mass of SnO_2@GC after sulfur removal. Also, note that the SnO_2@GCs with various original sulfur content have the same SnO_2 content of 67%, while the SnO_2@GCs with various SnO_2 content have the same original sulfur loading as SnO_2@GC-21.

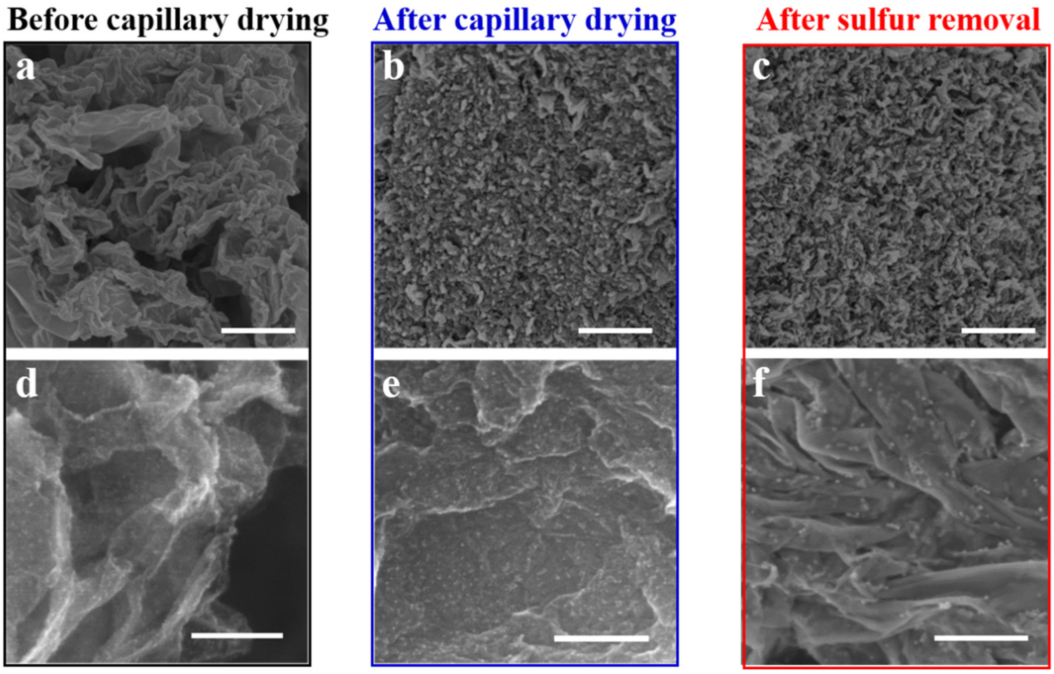

Supplementary Figure 2. Morphology change during the SnO_2@GC-21 synthesis process. SEM images of the sample **(a, d)** before capillary drying, **(b, e)** after capillary drying and **(c, f)** after sulfur removal process in low and high resolutions, respectively. Scale bars, **(a–c)** 1 μm; **(d–f)** 200 nm.

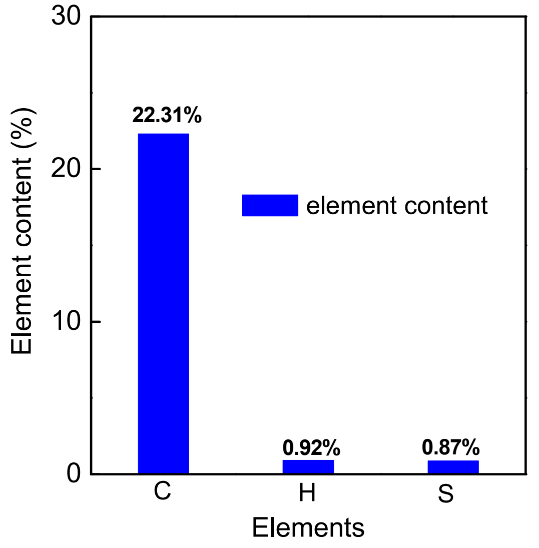

Supplementary Figure 3. CHNS analysis of C, H and S elements content in SnO_2@GC-21.

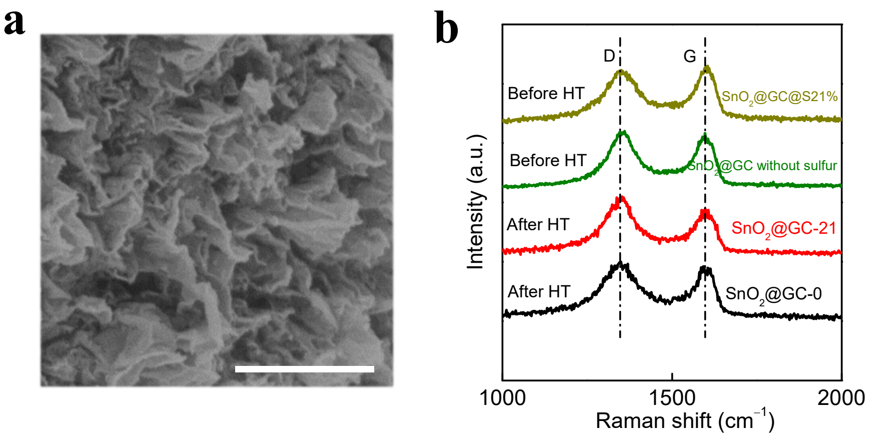

Supplementary Figure 4. **Morphology characterization of 3D graphene cage. (a)** SEM image of a 3D graphene cage after heat treatment (HT). **(b)** Raman spectra of SnO_2@GC-21 and SnO_2@GC-0 before and after 400 °C treatments (for SnO_2@GC-21, this is a sulfur removal process). Scale bar, **(a)** 500 nm.

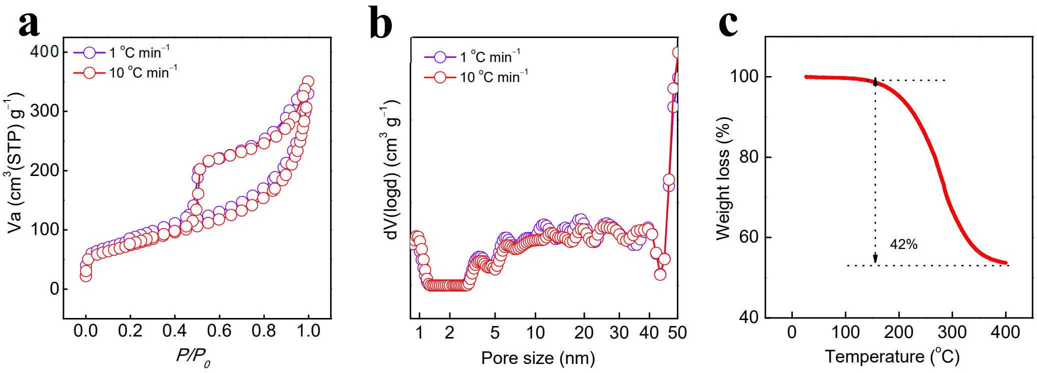

Supplementary Figure 5. **Influence of sulfur removal on pore structure of graphene cage. (a)** Nitrogen adsorption-desorption isotherms and **(b)** pore size distributions of GC@S (loading sulfur in the graphene cage free of SnO_2) subjected to the heat treatments (400 °C) with the heating rate of 1 °C min^{-1} and 10 °C min^{-1}, respectively. **(c)** TGA curve of GC@S. The preparation of GC@S was similar to that of the SnO_2@GC@S but free of the addition of $SnCl_4 \cdot 5H_2O$ as the precursor of SnO_2.

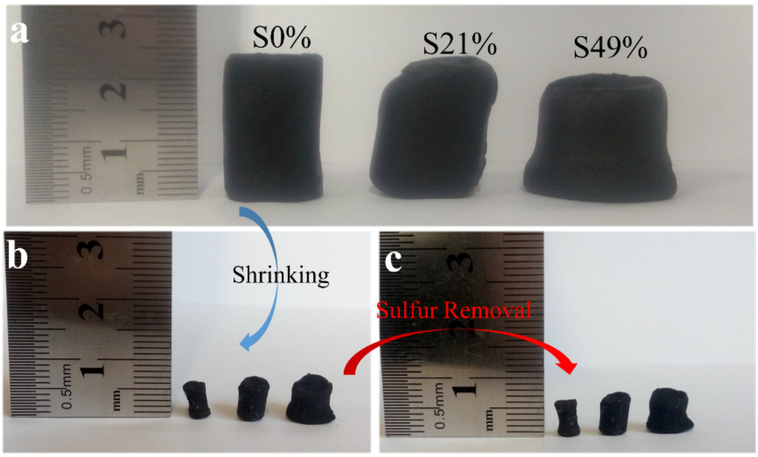

Supplementary Figure 6. **Volume changes of the SnO_2@GCs during capillary drying and sulfur removal process.** Photos of **(a)** hydrogels, **(b)** the capillary shrinkage-induced monolith of SnO_2@GC@S (before sulfur removal), and **(c)** the SnO_2@GCs (after sulfur removal).

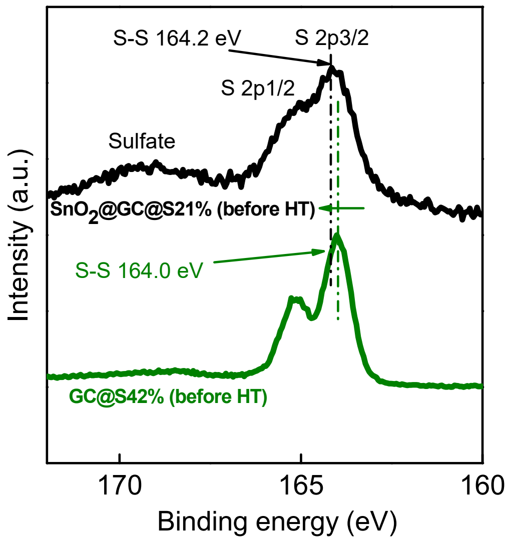

Supplementary Figure 7. S 2p XPS spectra with a 0.2 eV difference for S-S bond between GC@S42% (before HT) and SnO_2@GC@S21% (before HT).

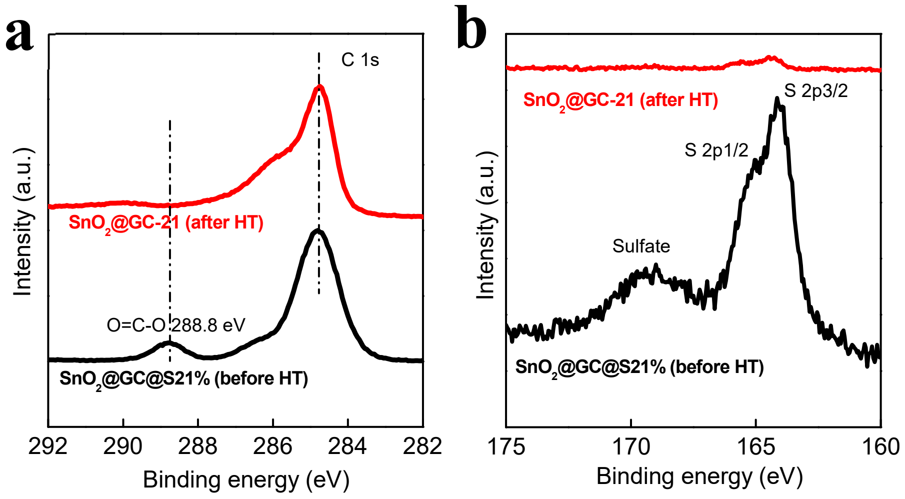

Supplementary Figure 8. C 1s and S 2p XPS spectra before and after sulfur removal process. **(a)** C 1s XPS spectra with O=C-O bond change, and **(b)** S 2p XPS spectra change after sulfur removal.

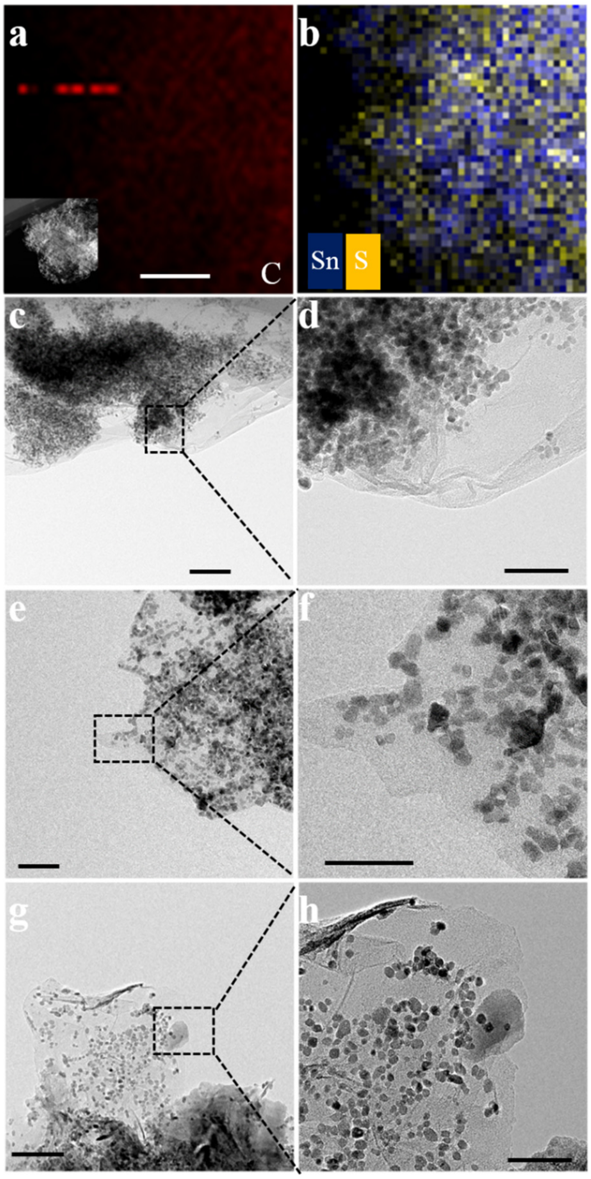

Supplementary Figure 9. **Void space change with various original sulfur content**. **(a, b)** STEM and EDS of SnO_2@GC-21 in a relatively low magnification. **(c, d)** TEM images of SnO_2 NPs in SnO_2@GC-0 without sulfur templating. SnO_2 NPs distribution in SnO_2@GC-21 **(e, f)** and -49 **(g, h)**. Scale bars, **(a)** 200 nm; **(c)** 100 nm; **(d)** 50 nm; **(e)** 100 nm; **(f)** 50 nm; **(g)** 100 nm; **(h)** 50 nm.

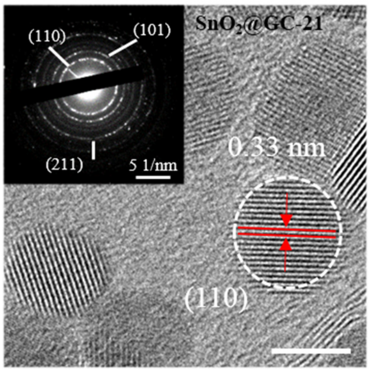

Supplementary Figure 10. HRTEM and SAED images of SnO_2@GC-21. Scale bar, 5 nm.

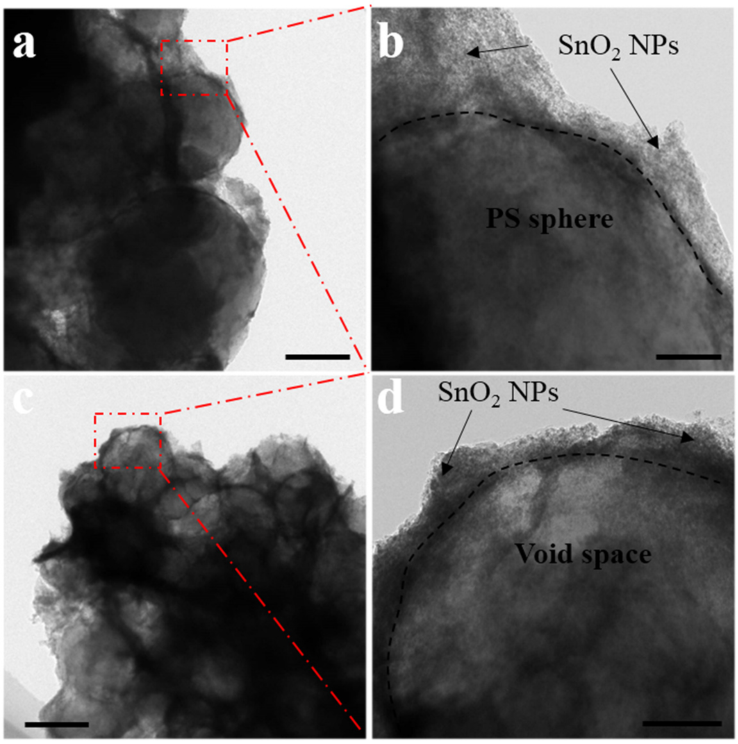

Supplementary Figure 11. **PS templated SnO_2@GC. (a–d)** TEM images of PS sphere templating in SnO_2@GC. Scale bars, **(a)** 500 nm; **(b)** 100 nm; **(c)** 500 nm; **(d)** 100 nm.

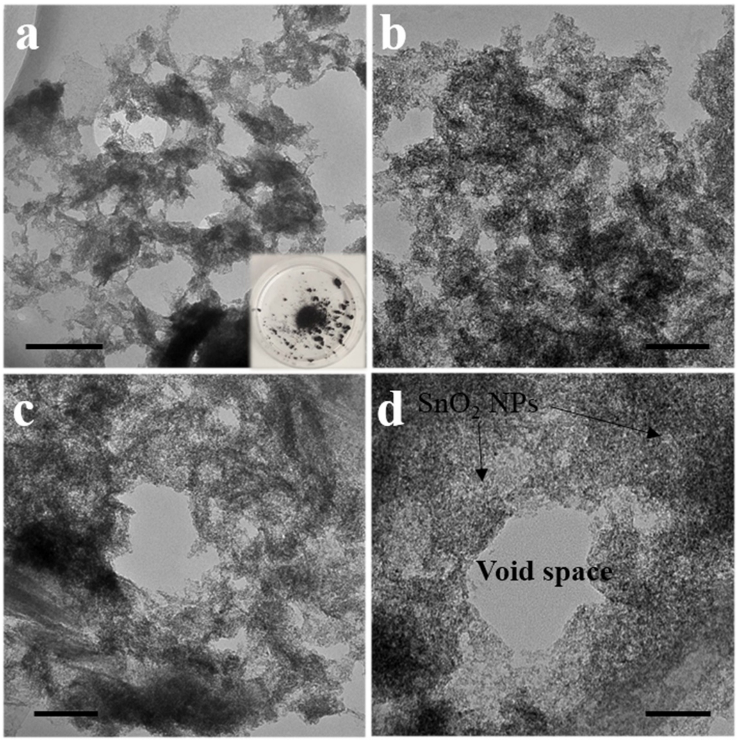

Supplementary Figure 12. **NaCl templated SnO_2@GC**. (**a–d**) TEM images of NaCl templating for void space incorporation. Scale bars, **(a)** 500 nm; **(b-c)** 200 nm; **(d)** 100 nm.

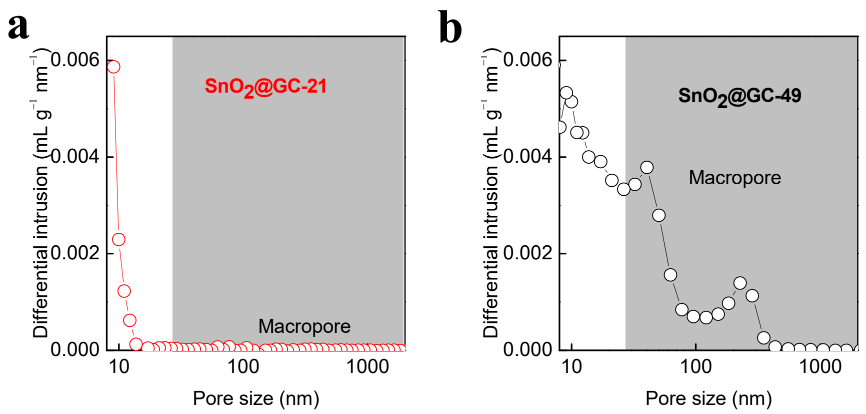

Supplementary Figure 13. Mercury intrusion porosimetry characterizations. Pore size distributions of SnO_2@GC-21 **(a)**, -49 **(b)** from Mercury intrusion porosimetry (MIP) analysis**.**

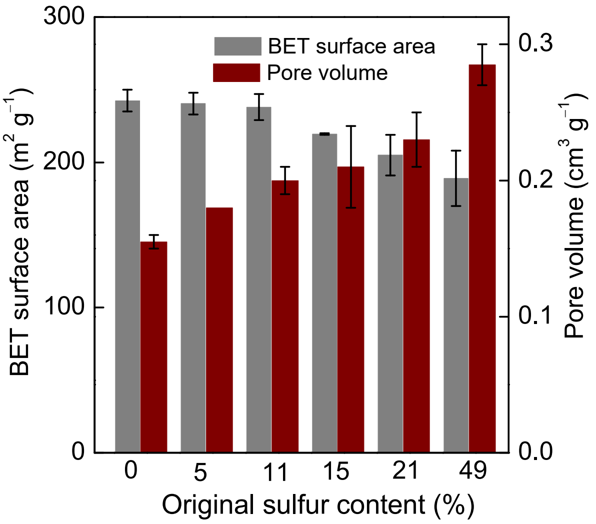

Supplementary Figure 14. The BET specific area and pore volume changes of the SnO_2@GCs with different content of sulfur as the removable templates. Error bars indicate s.d. (n = 3).

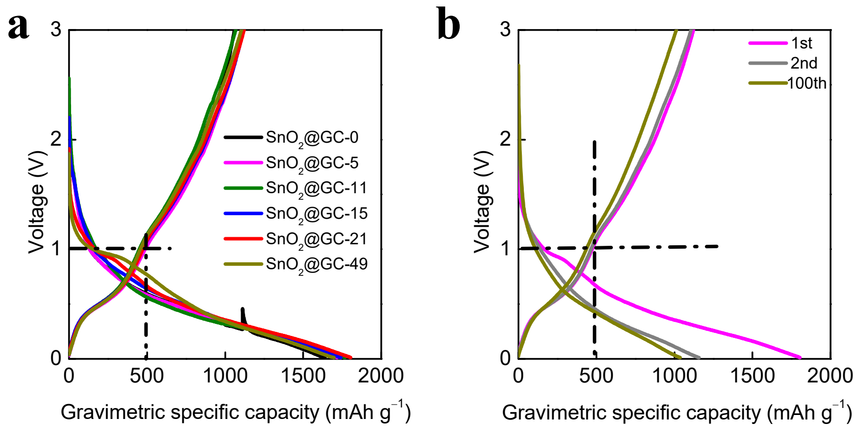

Supplementary Figure 15. **Galvanostatic charge/discharge characterizations.** Galvanostatic charge/discharge profiles of **(a)** the first cycle of SnO_2@GCs and **(b)** the first two and the 100th cycles of SnO_2@GC-21.

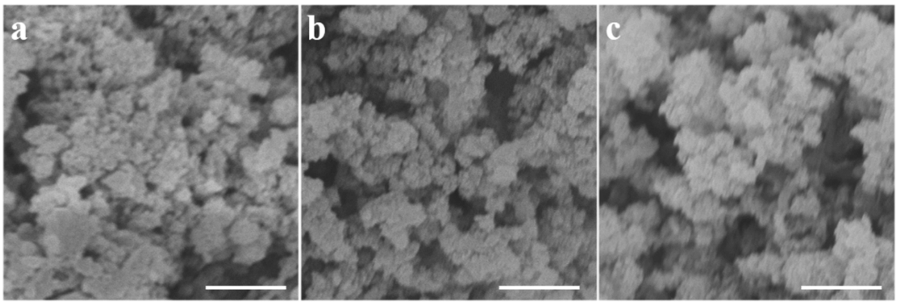

Supplementary Figure 16. **Electrode morphology of SnO_2@GCs after cycling.** SEM images of SnO_2@GC-0 **(a)**, -21 **(b)** and -49 **(c)** after 300 cycles at a current density of 100 mA g^{-1}. Scale bars, **(a–c)** 500 nm.

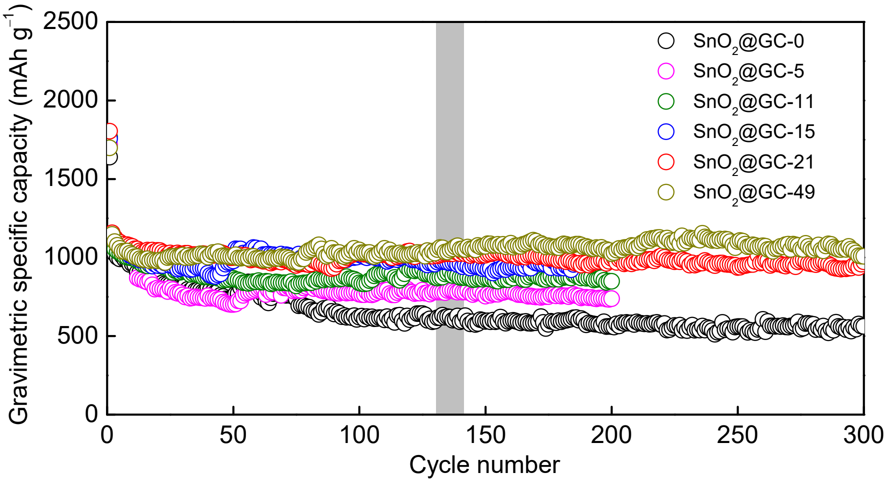

Supplementary Figure 17. Cycling performance of SnO_2@GCs at 100 mA g^{-1}.

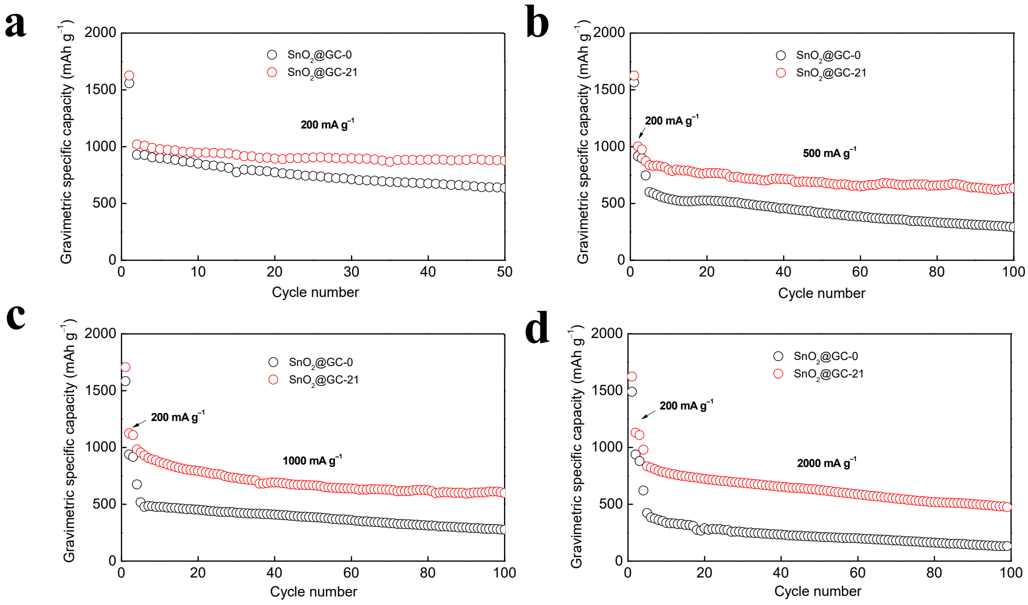

Supplementary Figure 18. Electrochemical performance with various C-rate. The cycling performance of SnO_2@GC-0 and -21 at different current densities of **(a)** 200 mA g^{-1}, **(b)** 500 mA g^{-1}, **(c)** 1000 mA g^{-1}, **(d)** 2000 mA g^{-1}.

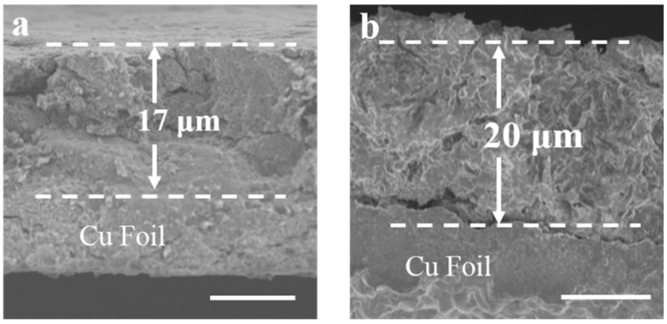

Supplementary Figure 19. Electrode thickness change after lithiation. (**a**) SEM image of the SnO_2@GC-21 electrode containing binder and carbon black. (**b**) SEM image of the SnO_2@GC-21 electrode upon lithiation. Scale bars, (**a–b**) 10 µm.

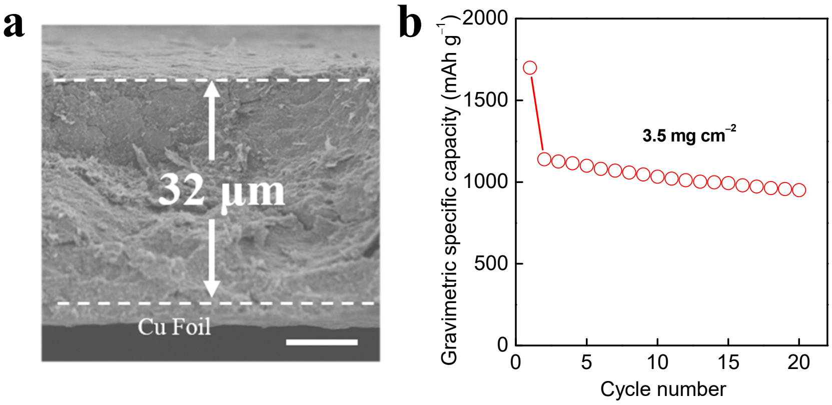

Supplementary Figure 20. Thick electrode characterizations. (a) Cross-sectional SEM image of thick SnO_2@GC-21 electrode morphology. **(b)** The cycling performance of SnO_2@GC-21 with a high mass loading of active materials at an areal current density of 0.2 mA cm^{-2}. Scale bar, (**a**) 10 µm.

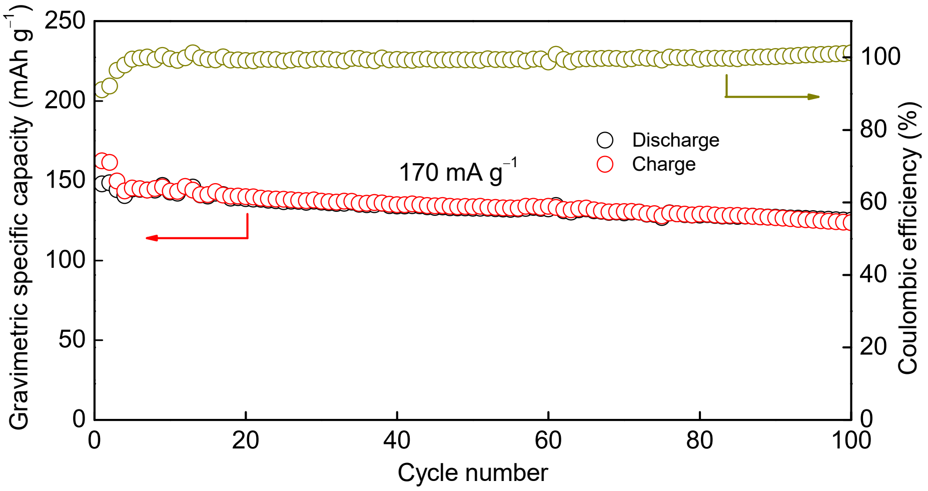

Supplementary Figure 21. Reversible capacity and Coulombic efficiency *versus* cycle plots of the LCO/SnO_2@GC-21 full cell with electrochemical pre-lithiation.

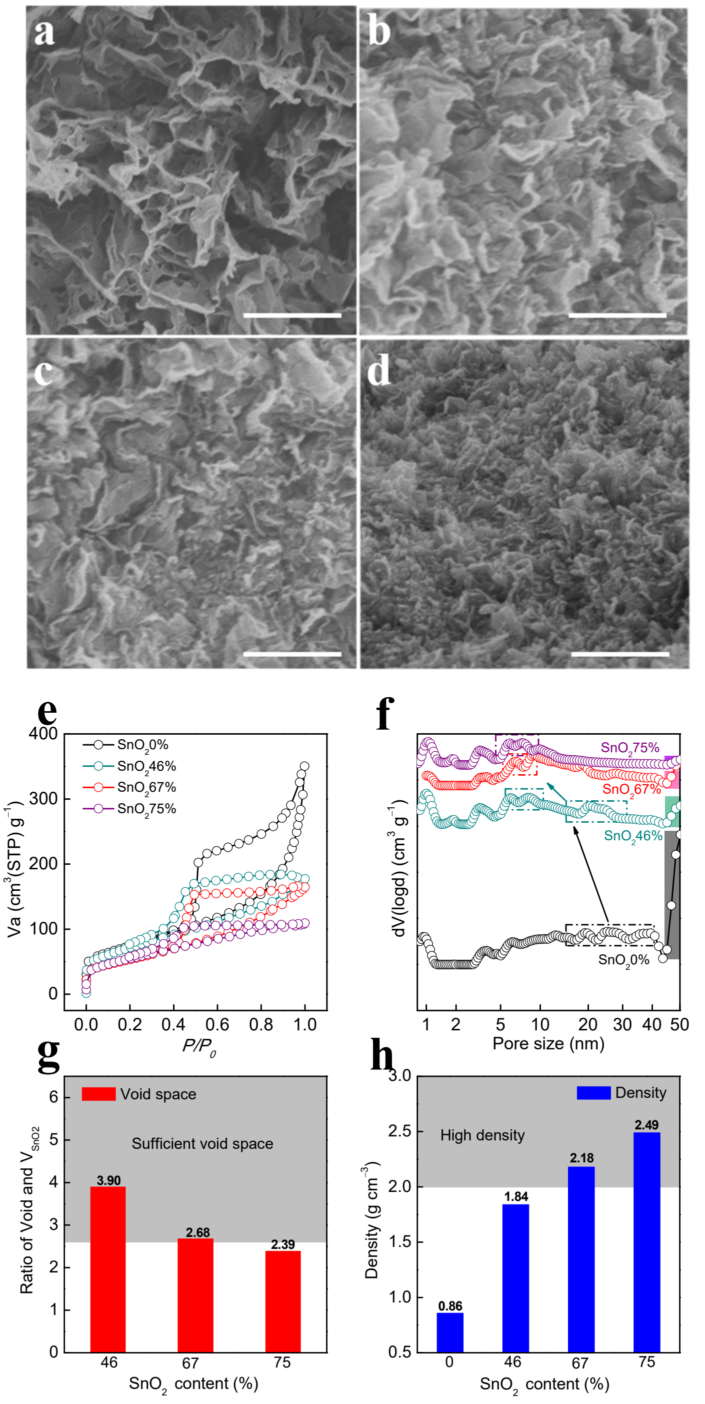

Supplementary Figure 22. **Structure change with various SnO_2 content. (a–d)** SEM images, **(e)** nitrogen adsorption-desorption isothermals, **(f)** pore size distributions, **(g)** calculated void spaces based on the SnO_2 volume, and **(h)** densities of SnO_2@GCs with different SnO_2 content of 0%, 46%, 67% and 75%. Scale bars, (**a–d**) 500 nm.

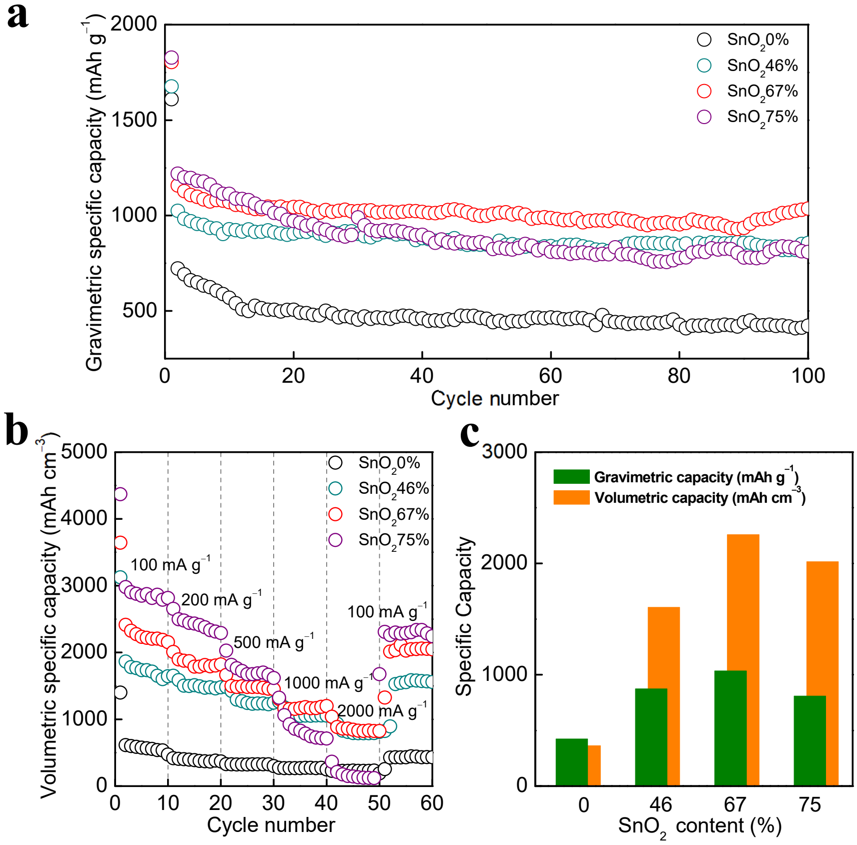

Supplementary Figure 23. **Electrochemical performance with various SnO_2 content. (a)** Cycling performance and **(b)** rate performance of SnO_2@GCs with different SnO_2 content of 0%, 46%, 67% and 75%. **(c)** Volumetric and gravimetric capacities of SnO_2@GCs with the SnO_2 content of 0%, 46%, 67% and 75%.

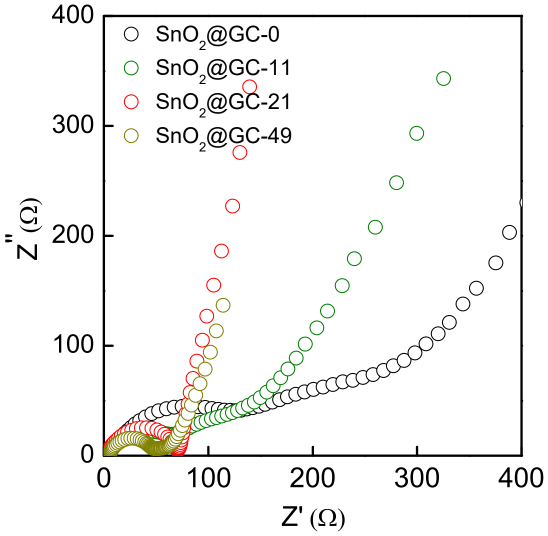

Supplementary Figure 24. Electrochemical impedance spectra of SnO_2@GCs before cycling.

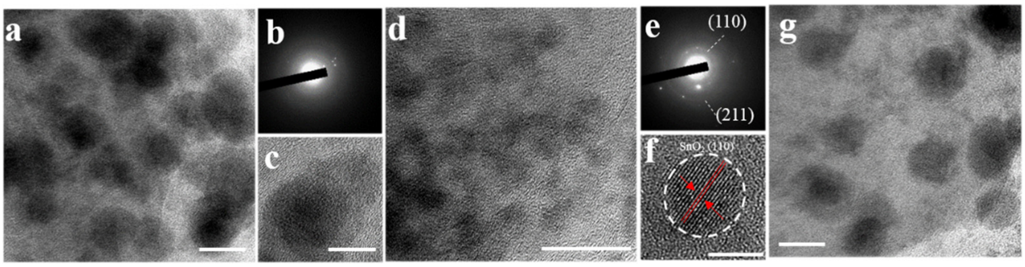

Supplementary Figure 25. ***Ex situ* TEM characterization of SnO_2 volume change after lithiation**. TEM and SAED images of (**a–c**) SnO_2@GC-21 and (**d–f**) SnO_2@GC-0 upon lithiation. **(g)** TEM image of SnO_2@GC-49 upon lithiation. Scale bars, **(a)** 20 nm; **(c)** 10 nm; **(d)** 20 nm; **(f)** 5 nm; **(g)** 20 nm.

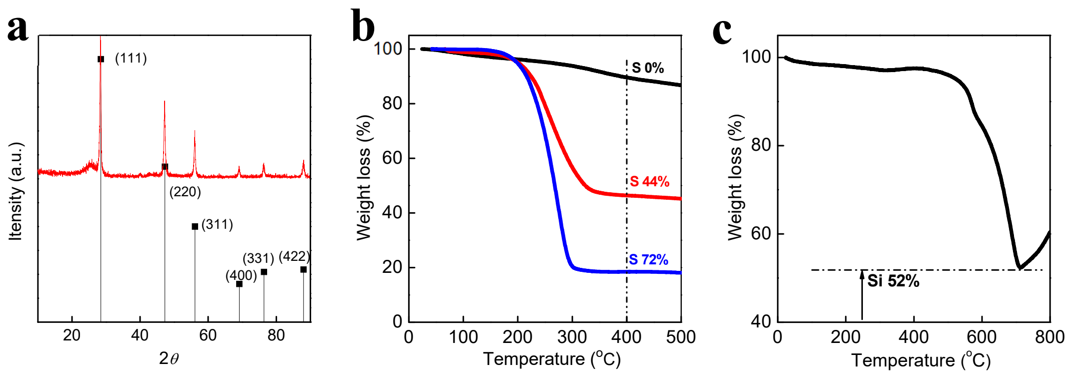

Supplementary Figure 26. **The existence and content of Si. (a)** XRD of Si@GC. **(b)** TGA curves of Si@GC-0, -44 and -72 with original sulfur content of 0%, 44% and 72%, respectively. **(c)** TGA curve of the Si@GC.

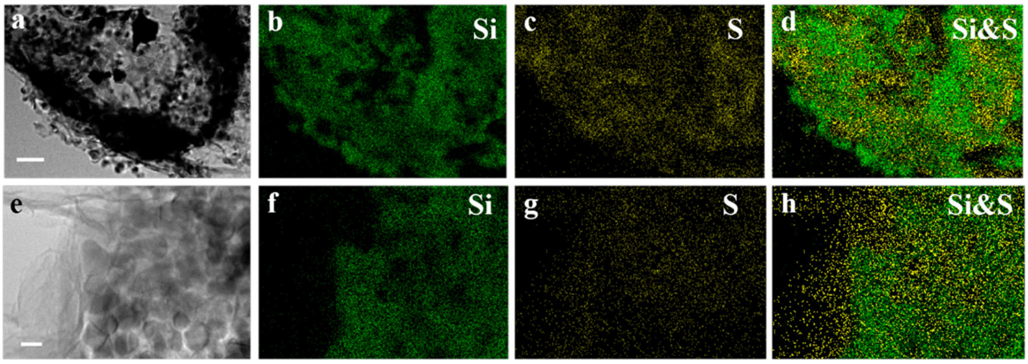

Supplementary Figure 27. The Si and S element distributions in Si@GC@S. EDS of the Si@GC@S44% in a low magnification **(a–d)** and a relatively high magnification **(e–h)**. Scale bars, (**a**) 200 nm; (**e**) 50 nm.

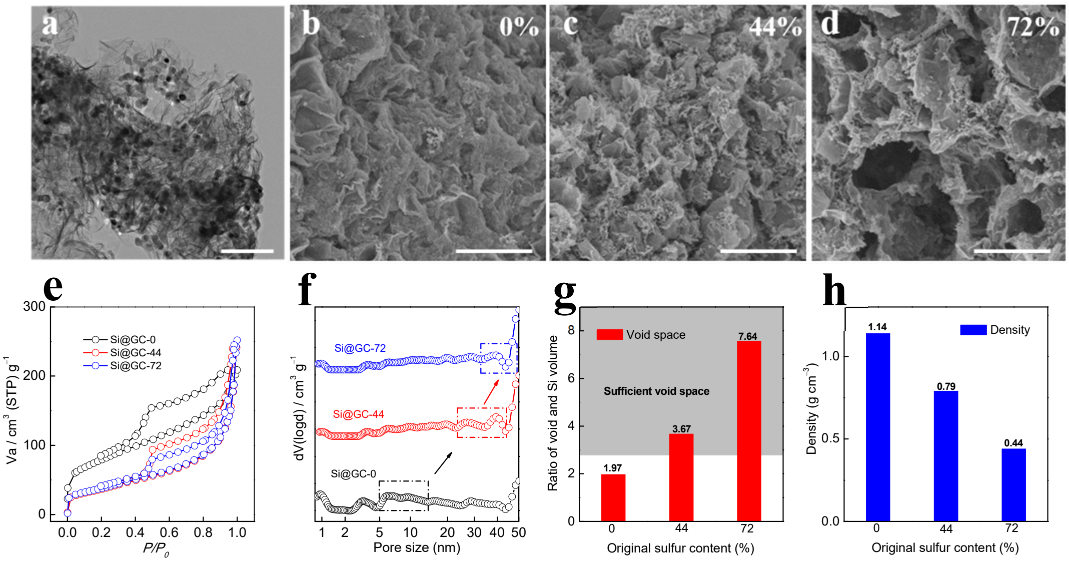

Supplementary Figure 28. Structure change of Si@GCs with various sulfur content. (a) TEM image of the Si NPs (~50 nm) distribution in the graphene cage. **(b–d)** SEM images of Si@GC-0, -44 and -72. **(e)** Nitrogen adsorption-desorption isotherms, **(f)** pore size distributions, **(g)** calculated void space and **(h)** density changes of Si@GCs. Scale bars, **(a)** 500 nm; **(b–d)** 5 μm.

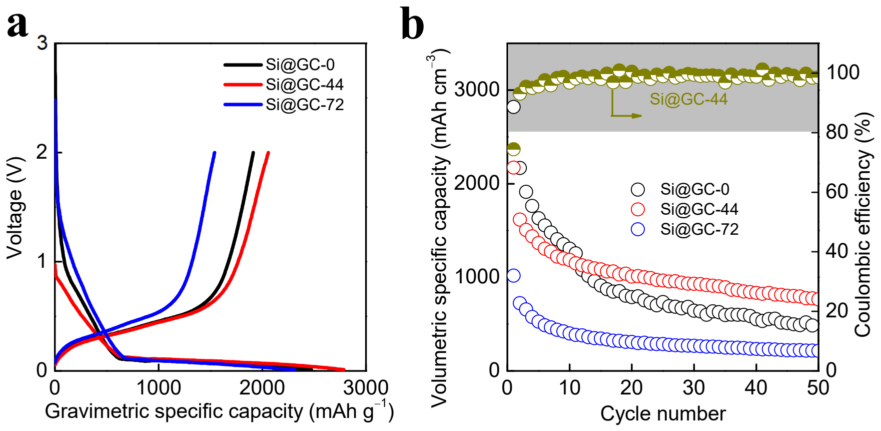

Supplementary Figure 29. Galvanostatic charge/discharge charaterizations. Galvanostatic charge/discharge profiles of **(a)** the first cycle of Si@GCs, **(b)** cycling performance of Si@GCs at 200 mA g^{-1}.

Supplementary Table 1. Comparison of volumetric capacity of SnO_2@GC-21 with the reported representative Si, Sn-based anodes in LIBs.

**Materials**	Density of active materials ($g\ cm^{-3}$)	Volumetric capacity based on the active materials ($mAh\ cm^{-3}$)	Density of electrode ($g\ cm^{-3}$)	Volumetric capacity based on the electrode ($mAh\ cm^{-3}$)	cycles	Current Density	Voltage	Active material mass loading ($mg\ cm^{-2}$)	Binder (content)	Ref
**SnO_2@GC-21**	2.18	2123	1.38	1075	300	100 $mA\ g^{-1}$	0.01–3 V	1.87	10% PVDF	This work
**PVP-Sn(IV)@Ti3C**	2.16	1374.8	N/A	N/A	50	100 $mA\ g^{-1}$	0.01–3 V	N/A	15% PVDF	1
**Fe_2O_3-G_2**	2.3	1403	1.3	634	100	200 $mA\ g^{-1}$	0.005–3 V	1.89	10% PVDF	2
**Sn/C nanocomposite**	1.92	1700	1.92	1700	200	0.5 C	0.01–3 V	N/A	binder-free	3
**NHGM**	1.1	1052	N/A	N/A	1200	0.1 $mA\ cm^{-2}$	0.01–3 V	2.75	5% PVDF	4
**The bowl-like SnO_2@C**	0.24	231	N/A	N/A	100	400 $mA\ g^{-1}$	0.005–3 V	N/A	10% PVDF	5
**t-Si@GN arrays**	1.0	1500	1.0	1500	200	840 $mA\ g^{-1}$	0.02–2 V	0.9	binder-free	6
**3D Si membrane**	0.167	429	0.167	429	100	300 $mA\ g^{-1}$	0.01–1.5 V	N/A	binder-free	7
**SiNP-PANi**	0.899	1078	0.899	1078	600	1000 $mA\ g^{-1}$	0.01–1 V	0.3	binder-free	8
**SiNW fabric**	0.233	116.5	0.233	116.5	100	179 $mA\ g^{-1}$	0.01–1 V	N/A	binder-free	9
**SSG**	1.926	1087	N/A	N/A	60	239 $mA\ g^{-1}$	0.01–3 V	2.29	3% CMC	10
**Si-C granule**	0.49	779	N/A	N/A	100	1 C	0–1.1 V	N/A	PVDF with 10 wt% PAA	11

**SG-Si-c-PAN**	N/A	N/A	0.85	2350	100	100 mA g^{-1}	0.05–1.5 V	1.3–2.5	PAN	12
**SiNW-PG**	N/A	N/A	1.5	1014	100	0.2 C	0.05–1.4 V	N/A	SBR and CMC (15%)	13
**FeO_x@C-2**	N/A	N/A	0.35	280	100	200 mA g^{-1}	0.01–3 V	N/A	10% PVDF	14
**Si pomegranate**	N/A	N/A	0.4	1270	1000	C/2	0.01–1 V	~ 0.2	10% PVDF	15
**SiNP-alginate**	N/A	N/A	0.5	850	100	4200 mA g^{-1}	0.01–1 V	N/A	sodium alginate	16
**nC-SiMP**	N/A	N/A	0.55	665	1000	C/4	0.01–1 V	~ 0.5	10% PVDF	17
**Si-SHP/CB (after volume expansion)**	N/A	N/A	1.1	1301	120	0.1 mA cm^{-2}	0.01–1 V	1.70	Self-healing polymer	18
**5wt%-Gr–Si**	N/A	N/A	N/A	2500	1	N/A	0.01–1.5 V	1.2	1.1M Li-polyacrylic acid	19
**Gr-Si-CNM**	~ 2.3	2821 (average)	~ 2.3	2821 (average)	1000	1800 mA g^{-1}	0.01–1.5 V	0.07	binder-free	20
**SF electrode**	N/A	N/A	N/A	1799	150	500 mA g^{-1}	0.02–1.0 V	0.9–1.5	sodium alginate	21
**h-SnO_2@Si-2**	N/A	N/A	1.33	1030	500	100 mA g^{-1}	0.01– ~1 V	1.9–2.0	N/A	22
**Fe_2N@C electrode**	N/A	N/A	N/A	1030 (lithiated)	50	100 mA g^{-1}	0.01–3 V	12 μm (lithiated)	N/A	23

Supplementary Table 2. The detailed experimental parameters for materials preparations.

**Sample**	**GO(mL)**	**$Na_2S_2O_3·5H_2O$(g)**	**HCl(mL)**	**$SnCl_4·5H_2O$(mg)**
**SnO_2@GC@S0%**	57	0	0	235
**SnO_2@GC@S5%**	57	0.09	0.68	235
**SnO_2@GC@S11%**	57	0.21	1.73	235
**SnO_2@GC@S15%**	57	0.37	3.19	235
**SnO_2@GC@S21%**	57	0.54	4.72	235
**SnO_2@GC@S49%**	57	3.41	28.0	235
**$SnO_2$0%@GC@S**	57	0.54	4.72	0
**$SnO_2$46%@GC@S**	57	0.54	4.72	117.5
**$SnO_2$67%@GC@S**	57	0.54	4.72	235
**$SnO_2$75%@GC@S**	57	0.54	4.72	470

**Sample**	**GO (mL)<br>(4 mg mL^{-1})**	**$Na_2S_2O_3·5H_2O$ (g)**	**HCl (mL)**	**Si (mg)<br>(Solved in 28.5 mL ethanol)**
**Si@GC@S44%**	28.5	1.27	10.29	57
**Si@GC@S72%**	28.5	3.41	28.00	57

**Sample**	**GO (mL)**	**PS suspension (mL)<br>(20 mg mL^{-1}, sizes of 500–1000 nm)**	**$SnCl_4·5H_2O$ (mg)**
**SnO_2@GC@PS**	57	2	235

**Sample**	**GO (mL)**	**NaCl (mL)<br>(4 M)**	**$SnCl_4·5H_2O$ (mg)**
**SnO_2@GC@NaCl**	57	50	235

Supplementary Table 3. The detailed results of calculated void spaces of SnO_2@GCs. ρ is the monolith densities of SnO_2@GC and Si@GC determined by Archimedes principle with a balance (Mettler Toledo XS205) equipped with accessories for the density determination; T_s and T_v are respectively the volume ratios of the original sulfur volume, void space to the SnO_2 volume in SnO_2@GC and Si@GC; ρ' and T_{v1} are respectively the calculated density and calculated void space of SnO_2@GC and Si@GC according to the equations of (2–4) in main text; T_{v2} is the volume ratio of the pore volume and the SnO_2 volume in SnO_2@GC. These above results demonstrate minor errors among experimental data and calculations for densities and voids in SnO_2@GCs and Si@GC. The differences between pore volume and the calculated void volume at a high sulfur content are mainly ascribed to the fact that the macropore volume is difficult to be ascertained by the N_2 adsorption-desorption measurement (the large pores over 500 nm in size cannot be identified by the N_2 adsorption-desorption isotherm).

	SnO_2@GC-0	SnO_2@GC-5	SnO_2@GC-11	SnO_2@GC-15	SnO_2@GC-21	SnO_2@GC-49
ρ (g cm^{-3})	2.68	2.51	2.32	2.25	2.18	1.16
T_s	0	0.26	0.62	0.88	1.33	4.81
T_v	1.35	1.61	1.97	2.23	2.68	6.16
ρ' (g cm^{-3})	2.68	2.51	2.31	2.18	2.00	1.19
T_{v1}	1.35	1.61	1.97	2.09	2.24	6.42
T_{v2}	1.56	1.87	2.07	2.18	2.39	2.96
	**Si@GC-0**	**Si@GC-44**	**Si@GC-72**			
ρ (g cm^{-3})	1.14	0.79	0.44			
T_s	0	1.70	5.67			
T_v	1.97	3.67	7.64			
ρ' (g cm^{-3})	1.14	0.79	0.47			
T_{v1}	1.97	3.72	8.23			

Supplementary References

1. Luo, J. M. *et al.* Sn^{4+} ion decorated highly conductive Ti_3C_2 Mxene: promising lithium-ion anodes with enhanced volumetric capacity and cyclic performance. *ACS Nano* **10**, 2491–2499 (2016).

2. Li, Z. *et al.* Twin-functional graphene oxide: compacting with Fe_2O_3 into a high volumetric capacity anode for lithium ion battery. *Energy Storage Mater.* **6**, 98–103 (2017).

3. Liu, J. Y. *et al.* High volumetric capacity three-dimensionally sphere-caged secondary battery anodes. *Nano Lett.* **16**, 4501–4507 (2016).

4. Wang, X. P. *et al.* High-density monolith of N-doped holey graphene for ultrahigh volumetric capacity of Li-ion batteries. *Adv. Energy Mater.* **6**, 1502100 (2016).

5. Liang, J. *et al.* Bowl-like SnO_2@carbon hollow particles as an advanced anode material for lithium-ion batteries. *Angew. Chem. Int. Ed.* **53**, 12803–12807 (2014).

6. Wang, B. *et al.* High volumetric capacity silicon-based lithium battery anodes by nanoscale system engineering. *Nano Lett.* **13**, 5578–5584 (2013).

7. Xia, F. *et al.* Facile synthesis of free-Standing silicon membranes with three-dimensional nanoarchitecture for anodes of lithium ion batteries. *Nano Lett.* **13**, 3340–3346 (2013).

8. Wu, H. *et al.* Stable Li-ion battery anodes by in-situ polymerization of conducting hydrogel to conformally coat silicon nanoparticles. *Nat. Commun.* **4**, 1943 (2013).

9. Chockla, A. M. *et al.* Silicon nanowire fabric as a lithium ion battery electrode material. *J. Am. Chem. Soc.* **133**, 20914–20921 (2011).

10. Yin, J. F., Cao, H. Q., Zhou, Z. F., Zhang, J. X. & Qu, M. Z. SnS_2@reduced graphene oxide nanocomposites as anode materials with high capacity for rechargeable lithium ion batteries. *J. Mater. Chem.* **22**, 23963–23970 (2012).

11. Magasinski, A. *et al.* High-performance lithium-ion anodes using a hierarchical bottom-up approach. *Nat. Mater.* **9**, 353–358 (2010).

12. Hassan, F. M. *et al.* Evidence of covalent synergy in silicon-sulfur-graphene yielding highly efficient and long-life lithium-ion batteries. *Nat. Commun.* **6**, 8597 (2015).

13. Jeong, S. *et al.* Etched graphite with internally grown Si nanowires from pores as an anode for high density Li-ion batteries. *Nano Lett.* **13**, 3403–3407 (2013).

14. Zhang, H. W. *et al.* Tailoring the void size of iron oxide@carbon yolk-shell structure for optimized lithium storage. *Adv. Funct. Mater.* **24**, 4337–4342 (2014).

15. Liu, N. *et al.* A pomegranate-inspired nanoscale design for large-volume-change lithium battery anodes. *Nat. Nanotechnol.* **9**, 187–192 (2014).

16. Kovalenko, I. *et al.* A major constituent of brown algae for use in high-capacity Li-ion batteries. *Science* **334**, 75–79 (2011).

17. Lu, Z. D. *et al.* Nonfilling carbon coating of porous silicon micrometer-sized particles for high-performance lithium battery anodes. *ACS Nano* **9**, 2540–2547 (2015).

18. Chen, Z. *et al.* High-areal-capacity silicon electrodes with low-cost silicon particles based on spatial control of self-healing binder. *Adv. Energy Mater.* **5**, 1401826 (2015).

19. Son, I. H. *et al.* Silicon carbide-free graphene growth on silicon for lithium-ion battery with high volumetric energy density. *Nat. Commun.* **6**, 7393 (2015).

20. Suresh, S. *et al.* Protecting silicon film anodes in lithium-ion batteries using an atomically thin graphene drape. *ACS Nano* **11**, 5051–5061 (2017).

21. Zhang, X. H. *et al.* Silicene flowers: a dual stabilized silicon building block for high-performance lithium battery anodes. *ACS Nano* **11**, 7476–7484 (2017).

22. Ma, T. Y. *et al.* High volumetric capacity of hollow structured SnO_2@Si nanospheres for lithium-ion batteries. *Nano Lett.* **17**, 3959–3964 (2017).

23. Dong, Y. F. *et al.* Air-stable porous Fe_2N encapsulated in carbon microboxes with high volumetric lithium storage capacity and a long cycle life. *Nano Lett.* **17**, 5740–5746 (2017).
