# Supplementary material for: Caging tin oxide in three-dimensional graphene networks for superior volumetric lithium storage
Source: Nat Commun. 2018 Jan 26;9:402. doi: 10.1038/s41467-017-02808-2 (PMC5786064; doi:10.1038/s41467-017-02808-2)
Supplement: Supplementary file 2 — Description of Additional Supplementary Files [file 41467_2017_2808_MOESM2_ESM.pdf]

Description of Additional Supplementary Files

File Name: Supplementary Movie 1
Description: Lithiation of SnO_2@GC-21 with appropriate void space to observe the void space utilization. The movie is played at 8x speed.

File Name: Supplementary Movie 2
Description: Lithiation of SnO_2@GC-21 with appropriate void space to observe the SnO_2 NPs expansion within the void of graphene network. The movie is played at 8x speed.

File Name: Supplementary Movie 3
Description: Lithiation of SnO_2@GC-21 with appropriate void space to observe the single SnO_2 nanoparticle transformation. The movie is played at 8x speed.

File Name: Supplementary Movie 4
Description: Lithiation of SnO_2@GC-0 with insufficient void space. The movie is played at 8x speed.

File Name: Supplementary Movie 5
Description: Lithiation of SnO_2@GC-49 with excess void space. The movie is played at 8x speed.
